# Diaphanous-related formin subfamily: Novel prognostic biomarkers and tumor microenvironment regulators for pancreatic adenocarcinoma

**DOI:** 10.3389/fmolb.2022.910950

**Published:** 2022-12-15

**Authors:** Bixi Zhang, Qing Hu, Yanchun Li, Canxia Xu, Xiaoran Xie, Peng Liu, Meihua Xu, Siming Gong, Hao Wu

**Affiliations:** ^**1**^ Department of Pathology, Hunan Provincial People’s Hospital, Hunan Normal University, Changsha, China; ^2^ Department of Gastroenterology, Third Xiangya Hospital, Central South University, Changsha, China; ^3^ Department of Gastroenterology, Xiangya Hospital, Central South University, Changsha, China; ^4^ Institute of Anatomy, University of Leipzig, Leipzig, Germany; ^5^ Center for Precision Medicine, University of Missouri School of Medicine, Columbia, MO, United States

**Keywords:** DIAPH1, DIAPH2, DIAPH3, pancreatic adenocarcinoma, prognosis, immunotherapy

## Abstract

The diaphanous-related formin subfamily includes diaphanous homolog 1 (DIAPH1), DIAPH2, and DIAPH3. DIAPHs play a role in the regulation of actin nucleation and polymerization and in microtubule stability. DIAPH3 also regulates the assembly and bipolarity of mitotic spindles. Accumulating evidence has shown that DIAPHs are anomalously regulated during malignancy. In this study, we reviewed The Cancer Genome Atlas database and found that DIAPHs are abundantly expressed in pancreatic adenocarcinoma (PAAD). Furthermore, we analyzed the gene alteration profiles, protein expression, prognosis, and immune reactivity of DIAPHs in PAAD using data from several well-established databases. In addition, we conducted gene set enrichment analysis to investigate the potential mechanisms underlying the roles of DIAPHs in the carcinogenesis of PAAD. Finally, we performed the experimental validation of DIAPHs expression in several pancreatic cancer cell lines and tissues of patients. This study demonstrated significant correlations between DIAPHs expression and clinical prognosis, oncogenic signature gene sets, T helper 2 cell infiltration, plasmacytoid dendritic cell infiltration, myeloid-derived suppressor cell infiltration, ImmunoScore, and immune checkpoints in PAAD. These data may provide important information regarding the role and mechanisms of DIAPHs in tumorigenesis and PAAD immunotherapy.

## Introduction

Diaphanous-related formins are conserved cellular elements linking signal transduction pathways with both the microfilament and microtubule-based cytoskeleton (Chesarone et al., 2010). Mammals possess three members of this protein family, diaphanous homolog 1 (DIAPH1), DIAPH2, and DIAPH3, which have been implicated in both normal cell physiology and tumor progression. Lynch and others first identified DIAPH1 and DIAPH2 in 1997 (Lynch et al., 1997). DIAPH1 plays a role in the regulation of actin nucleation and polymerization (Lammers et al., 2008). Rho acts on DIAPH1 to produce straight actin filaments and aligns microtubules (Yamana et al., 2006). DIAPH2 plays an essential role in achieving stable kinetochore microtubule attachment, regulated by Aurora B phosphorylation (Cheng et al., 2011). Katoh identified the human DIAPH3 gene in 2004 (Katoh and Katoh, 2004). DIAPH3 has similar functions in the regulation of the remodeling cytoskeleton as DIAPH1 and DIAPH2 (Palazzo et al., 2001), and it also regulates the assembly and bipolarity of the mitotic spindle (Lau et al., 2021).

DIAPH1, DIAPH2, and DIAPH3 (DIAPHs) are aberrantly regulated during malignancy. DIAPH1 is an essential regulator of tissue shape change and initiating invasion *via* the epithelial tissues (Fessenden et al., 2018). DIAPH1 expression is increased in patients with colorectal cancer, and its downregulation strongly reduces the metastatic capacity of colon carcinoma cells (Lin et al., 2014). Knockdown of DIAPH1 also inhibits the migration of human glioma cells (Zhang et al., 2017). DIAPH2 and DIAPH3 are required for invadopodia formation and tumor cell invasion (Lizárraga et al., 2009). DIAPH2 alterations increase cellular motility and may contribute to the onset and metastasis of laryngeal cancer (Kostrzewska-Poczekaj et al., 2019; Śnit et al., 2021). DIAPH3 is highly expressed in tissues of patients with pancreatic cancer, lung adenocarcinoma, and hepatocellular carcinoma (Dong et al., 2018; Xiang et al., 2019; Rong et al., 2021). A recent study demonstrated that paracrine activin A-DIAPH3 axis promotes squamous carcinogenesis *via* fibroblast reprogramming (Cangkrama et al., 2020). Indeed, DIAPH3 regulates the function of mitochondria in dermal fibroblasts, promoting a protumorigenic cancer-associated fibroblasts phenotype (Cangkrama et al., 2022). Pancreatic adenocarcinoma (PAAD) is prone to distant metastasis and remains a leading cause of cancer-related deaths worldwide (Klein, 2021). The underlying mechanism(s) for the role of DIAPHs in PAAD remain unelucidated. We reviewed The Cancer Genome Atlas (TCGA) database and found that DIAPHs are abundantly expressed in PAAD. Thus, bioinformatics analysis of DIAPHs may provide new insights into the molecular mechanisms underlying the occurrence, recurrence, and immunotherapy of PAAD.

In the present study, we analyzed the profiles of gene alterations, protein expression, prognosis, and immune reactivity of DIAPHs in PAAD based on several well-established databases. Furthermore, we conducted a DIAPHs-related gene enrichment analysis to investigate the mechanisms underlying the role of DIAPHs in PAAD. In addition, we performed western blotting of DIAPHs in several pancreatic cancer cell lines and immunohistochemical staining of DIAPH1, DIAPH2, DIAPH3, CD3, CD4, and CD68 in human pancreatic tumor tissues for experimental validation. This study aimed to explore the roles and mechanisms of DIAPHs in PAAD progression.

## Materials and methods

### RNA level expression analysis in pan-cancer

Pan-cancer RNA-Seq data were downloaded from the “TCGA TARGET GTEx” cohort of the UCSC Xena (https://xenabrowser.net/datapages/, accessed on 16 January 2022) and were processed by Toil recompute to create a consistent meta-analysis of TCGA cohorts and GTEx cohorts (Vivian et al., 2017). Using the rma function in the R package, DIAPHs mRNA-seq data were transformed with log2 (TPM + 1).

### Genetic and protein expression analysis in PAAD

The PAAD genetic alteration characteristics of DIAPHs based on TCGA database and four selected studies (Biankin et al., 2012; Witkiewicz et al., 2015; Bailey et al., 2016; Cao et al., 2021) were queried from cBioportal (https://www.cbioportal.org/, accessed on 18 January 2022) (Gao et al., 2013). The expression of DIAPHs between PAAD tumor tissues and normal tissues was further carried out through the “Box Plot” module of Gene Expression Profiling Interactive Analysis, version2 (GEPIA2) (http://gepia2.cancer-pku.cn/, accessed on 18 January 2022) (Tang et al., 2019). UALCAN (http://ualcan.path.uab.edu/analysis-prot.html, accessed on 18 January 2022) was used to conduct DIAPHs protein expression analysis in the Clinical Proteomic Tumor Analysis Consortium (CPTAC) Confirmatory/Discovery dataset (Chen et al., 2019).

### Survival and receiver operating characteristic analysis

The log-rank test was applied to the hypothesis test, and survival plots were obtained using the Kaplan-Meier “Survival Analysis” module of GEPIA2. Nomograms are widely used for cancer prognosis (Iasonos et al., 2008). Using clinical and biological variables, such as age, gender, histologic grade, and expression levels of DIAPHs, the intention was to graphically depict a prognostic model that generates the overall survival of PAAD. The R packages, “rms” and “survival” in the R software were used to perform prognostic nomograms (Liu et al., 2018). In addition, the R package “pROC” was used to perform receiver operating characteristic (ROC) curves (Robin et al., 2011). ROC curves are used to determine the diagnostic value of DIAPHs for PAAD.

### Protein-protein interactions and similar genes in PAAD

Using the STRING tool (https://string-db.org/, accessed on 20 January 2022), protein–protein interaction (PPI) analyses were performed with 50 available experimentally determined proteins that individually interacted with DIAPH1, DIAPH2, and DIAPH3, and these proteins were visualized in the PPI network. Based on the PAAD data from TCGA cohort, the top 100 DIAPH1-, DIAPH2-, and DIAPH3-correlated and -targeting genes were obtained from the “Similar Genes Detection” module of GEPIA2. Using the Venn diagram, we conducted intersection analyses to compare the DIAPHs-interacting and DIAPHs-correlated genes. After combining the two datasets, the R package “clusterProfiler” was used to perform the Kyoto Encyclopedia of Genes and Genomes (KEGG) pathway analysis and the Gene Ontology (GO) enrichment analysis, which included the biological processes (BP), the molecular functions (MF), and the cellular components (CC) (Yu et al., 2012). The intersected genes (if any) or the expression of the top five selected genes in PAAD are shown in a heatmap. In addition, a Spearman’s correlation test was performed on the intersected genes or top five selected genes using the “Gene_Corr” module of the TIMER2 website to obtain a pan-cancer heatmap.

### Gene set enrichment analysis

To explore the biological and oncogenic signaling, gene set enrichment analysis (GSEA) was performed on the low- and high-expression groups based on the mean expression values of DIAPHs in PAAD of TCGA database. The R package “clusterProfiler” was used to perform C6 (oncogenic signature gene sets) and MSigDB H (hallmark gene sets) enrichment analyses. The gene sets with |NES| > 1, FDR <0.25, and p. adjust <0.05, were considered to be significantly enriched.

### Immune reactivity analysis

The R package, “GSVA”, was used to perform the correlation between DIAPHs expression and infiltration of 24 common immune cells in PAAD of TCGA dataset by single-sample GSEA (ssGSEA) (Bindea et al., 2013; Hänzelmann et al., 2013). The correlation between DIAPHs expression and infiltration of myeloid-derived suppressor cells (MDSCs) using the TIDE algorithm in PAAD of TCGA database was obtained from the TIMER2 website. The R package “estimate” was used to perform the ImmunoScore between low- and high-expression groups based on the median expression values of DIAPHs in PAAD of TCGA dataset (Yoshihara et al., 2013). The SangerBox (http://sangerbox.com/, accessed on 22 January 2022) online platform was used to explore the relationship between DIAPHs expression and various immune checkpoints. Cellular heterogeneity of DIAPH3 expression in PAAD was conducted using Cancer Single-cell Expression Map (https://ngdc.cncb.ac.cn/cancerscem/index, last accessed on 1 April 2022) (Zeng et al., 2022).

### Cell culture and western blotting

HPDE-6, MIAPACA-2, and CAPAN-1 are purchased from BeNa Culture Collection (Beijing, China). HPDE-6 cells were cultured in RPMI-1640 supplemented with 10% FBS and 1% Penicillin-Streptomycin. MIAPACA-2 cells were cultured in DMEM supplemented with 10% FBS, 2.5% human serum, and 1% Penicillin-Streptomycin. CAPAN-1 were cultured in IMDM supplemented with 20% FBS and 1% Penicillin-Streptomycin. All the cells were cultured in a 37°C humidified incubator with a 5% CO2 environment.

Western blot the proteins through an SDS-PAGE gel and subsequently transferring them to 0.45 μm PVDF membrane (Merck). The membrane was blocked with 5% milk for 1 h, and then incubated with the primary antibody (DIAPH1: 1:5000 dilution, ab129167, Abcam; DIAPH2: 1:10,000 dilution, HPA005647, Sigma-Aldrich; DIAPH3: 1:5000 dilution, ab189373, Abcam; β-Tubulin: 1:1000 dilution, 10068-1-AP, Proteintech) overnight at 4°C. After three washes with TBST (each for 6 min), the membrane was incubated with the appropriate secondary antibodies at room temperature for 1 h, washed with TBST, and visualized using the ECL by ChemoStudio Imaging System (Analytik Jena).

### Immunohistochemical staining of pancreatic tissue samples

The pancreatic tissue samples used in this study were obtained from patients who underwent surgical treatment and diagnosed with pancreatic ductal adenocarcinoma at Hunan Provincial People’s Hospital (*n* = 5) and all patients provided written informed consent. The present study was conducted in accordance with the Declaration of Helsinki, and all experiments were approved by the ethics committees of Hunan Provincial People’s Hospital. Tissues were subjected to standard tissue processing and paraffin embedding. The tissues were sliced serially into sections 3 μm thick for hematoxylin and eosin (H&E) staining as described (Fischer et al., 2008). For immunohistochemical staining (IHC), the tissue sections were preheated in Tris-EDTA buffer (pH 9.0) and then maintain at a sub-boiling temperature for 20 min to retrieve the immunoreactivities of antigens. To block endogenous peroxidase activity, quench the tissue sections with 3.0% hydrogen peroxide at room temperature for 10 min. Antibodies against DIAPH1(1:800 dilution, ab129167; Abcam, United Kingdom), DIAPH2(1:1000 dilution, HPA005647; Sigma-Aldrich, United States), DIAPH3 (1:50 dilution, ab189373; Abcam, United Kingdom), CA19-9 (MAB-0778; MXB Biotechnologies, China), CK7 (KIT-0021; MXB Biotechnologies, China), CD3 (MAB-0740; MXB Biotechnologies, China), CD4 (RMA-0620; MXB Biotechnologies, China), and CD68 (KIT-0026; MXB Biotechnologies, China) were used to incubate the tissue sections for 1 h at room temperature. Sections were incubated with MaxVision HRP-Polymer anti-Mouse/Rabbit IHC Kit (KIT-5030, MXB Biotechnologies, China) for 30 min. The working solution of DAB (DAB-2032, MXB Biotechnologies, China) was applied to the tissue sections for the chromogenic reaction. The tissue sections were examined using an upright microscope (Eclipse Ni-U, Nikon Instruments, Japan).

### Statistical analysis

Gene expression data from TCGA and GTEx datasets were evaluated using the Wilcoxon signed-rank test. The protein expression data from the UALCAN database and western blotting were analyzed using Student’s *t*-test. Survival data from the GEPIA2 dataset were analyzed using the log-rank test. R software (Version 4.1.1) was used to perform prognostic nomograms, ROC curves, ImmunoScore, GO, KEGG pathway, MSigDB H, and C6 enrichment analyses. Correlation analysis was performed in the TIMER2 database using a purity-adjusted Spearman’s rho. Correlation analysis of immune checkpoints was performed using Pearson’s correlation coefficients. A *p* value less than 0.05 was considered to be statistically significant.

## Results

### Gene expression data in pan-cancer

**FIGURE 1:**
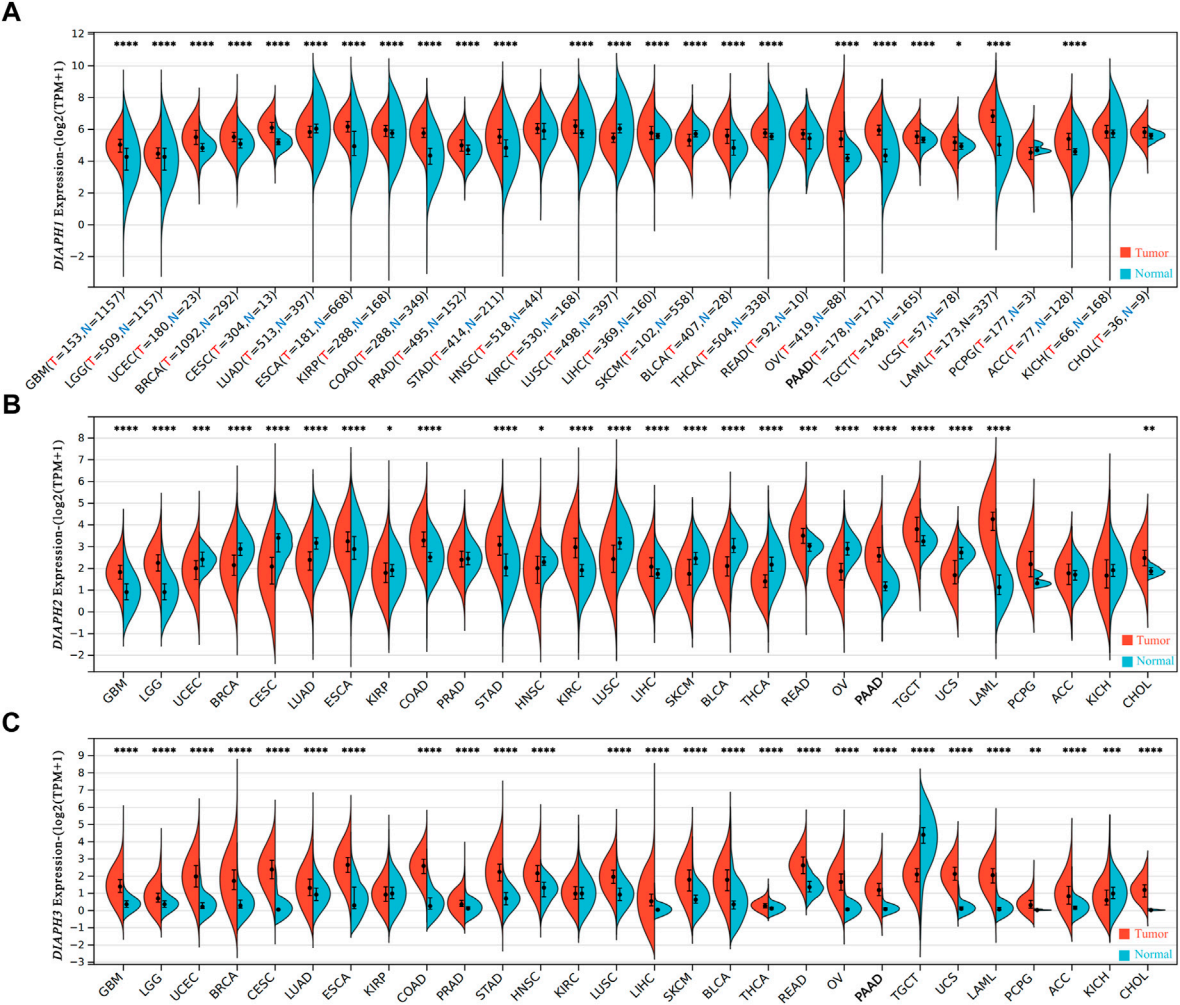
mRNA expression levels of DIAPHs in pan-cancer. **(A)** DIAPH1 expression. **(B)** DIAPH2 expression. **(C)** DIAPH3 expression. Log_2_ (TPM+1) transformed the expression data for plotting. *p < 0.05, **p < 0.01, ***p < 0.001, ****p < 0.0001, in Wilcoxon test. TPM, transcripts per million; N and T, normal and tumor tissues.

Analysis of the expression data of DIAPHs in different tumor tissues and normal tissues in the consensus datasets TCGA and GTEx showed that the expression levels of DIAPH1 and DIAPH3 were significantly higher in tumor tissues than in normal tissues across most of the tumors in TCGA database (Figures 1A,C), while the expression levels of DIAPH2 were significantly lower in tumor tissues than in normal tissues across different types of cancers (Figure 1B). Notably, the expression levels of DIAPH1, DIAPH2, and DIAPH3 were significantly higher in PAAD tumor tissues than in normal tissues.

### Gene expression data in PAAD

**FIGURE 2:**
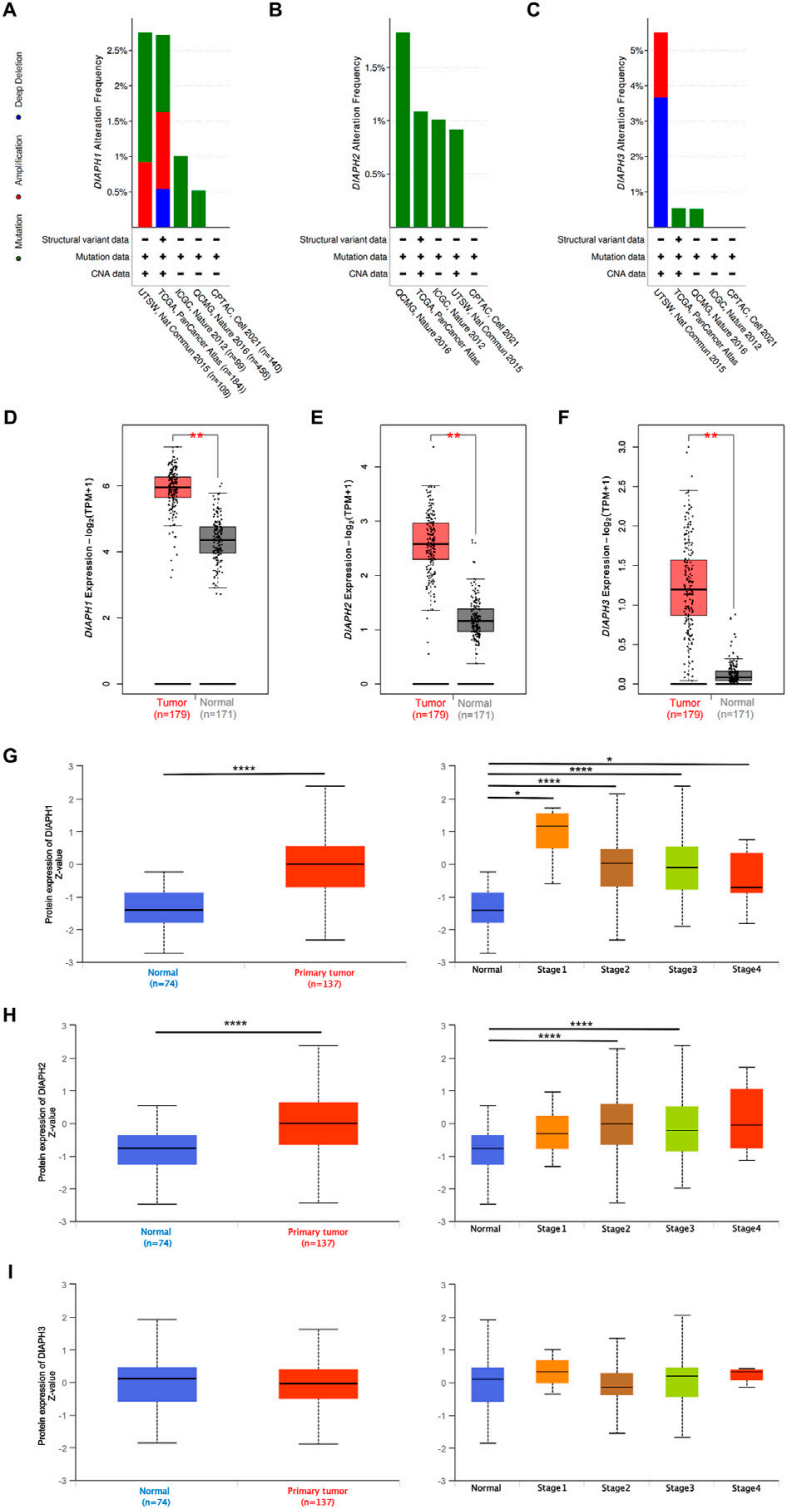
DIAPHs expression data in PAAD. **(A–C)** The PAAD genetic alteration characteristics of DIAPHs. **(D–F)** mRNA expression levels of DIAPHs in PAAD. Log_2_ (TPM+1) transformed the expression data for plotting. ** *p* < 0.01, in Wilcoxon test. **(G–I)** Total protein expression levels of DIAPHs in PAAD. Z values represent standard deviations from the median across samples. * *p* < 0.05, **** *p* < 0.0001, in Student’s *t* test. TPM, transcripts per million.

**FIGURE 3:**
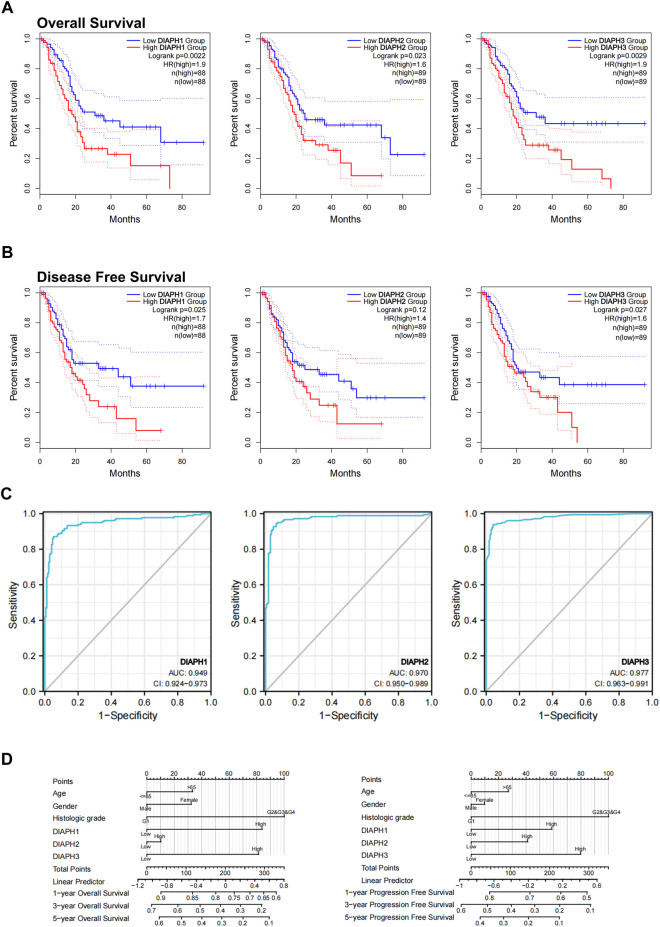
Relations between DIAPHs expression and prognosis of PAAD in TCGA database. **(A–B)** Overall survival and disease-free survival analyses between the DIAPHs high-expression and low-expression cohort. High-cutoff (50%) and low-cutoff (50%) values were used as the expression thresholds for separating the high-expression and low-expression cohorts, in log-rank test. HR, hazard ratio. **(C)** AUC of DIAPH1, DIAPH2, and DIAPH3 expression for PAAD. AUC, area under ROC curve. **(D)** DIAPHs as risk factors in nomograms for overall survival and progression-free survival in patients with PAAD.

The genetic alteration profiles of PAAD in TCGA dataset and four selected studies showed that 1.1% of the enrolled patients had DIAPH1 genetic alterations, 1.1% had DIAPH2 genetic alterations (predominantly missense mutations), and 0.9% had DIAPH3 genetic alterations (Figures 2A–C). The expression levels of DIAPH1, DIAPH2, and DIAPH3 in PAAD tumor tissues were significantly higher than in normal tissues in the consensus databases of TCGA and GTEx (Figures 2D–F). In the CPTAC database, the total DIAPH1 and DIAPH2 protein expression levels were higher in PAAD tumor tissues than in normal tissues (Figures 3G,H), whereas DIAPH3 protein expression did not differ between PAAD tumor tissues and normal tissues (Figure 3I).

### Survival and ROC analysis

Based on the expression levels of DIAPHs in PAAD, the correlation between DIAPHs expression and the prognoses of PAAD was explored in TCGA database. Increased levels of DIAPH1, DIAPH2, and DIAPH3 were significantly associated with poor overall survival and disease-free survival (Figures 3A,B). The area under the ROC curve (AUC) of DIAPH1, DIAPH2, and DIAPH3 expression for PAAD was 0.949, 0.970, and 0.977, respectively (Figure 3C). Nomograms for overall survival and progression-free survival in patients with PAAD showed that high DIAPHs expression were risk factors (Figure 3D). In addition, the baseline TNM stages and age in patients with PAAD did not differ between the low- and high-expression groups based on the median expression values of DIAPHs (Supplementary Tables S1–S3).

### Protein-protein interactions of DIAPHs and similar genes in PAAD

**FIGURE 4:**
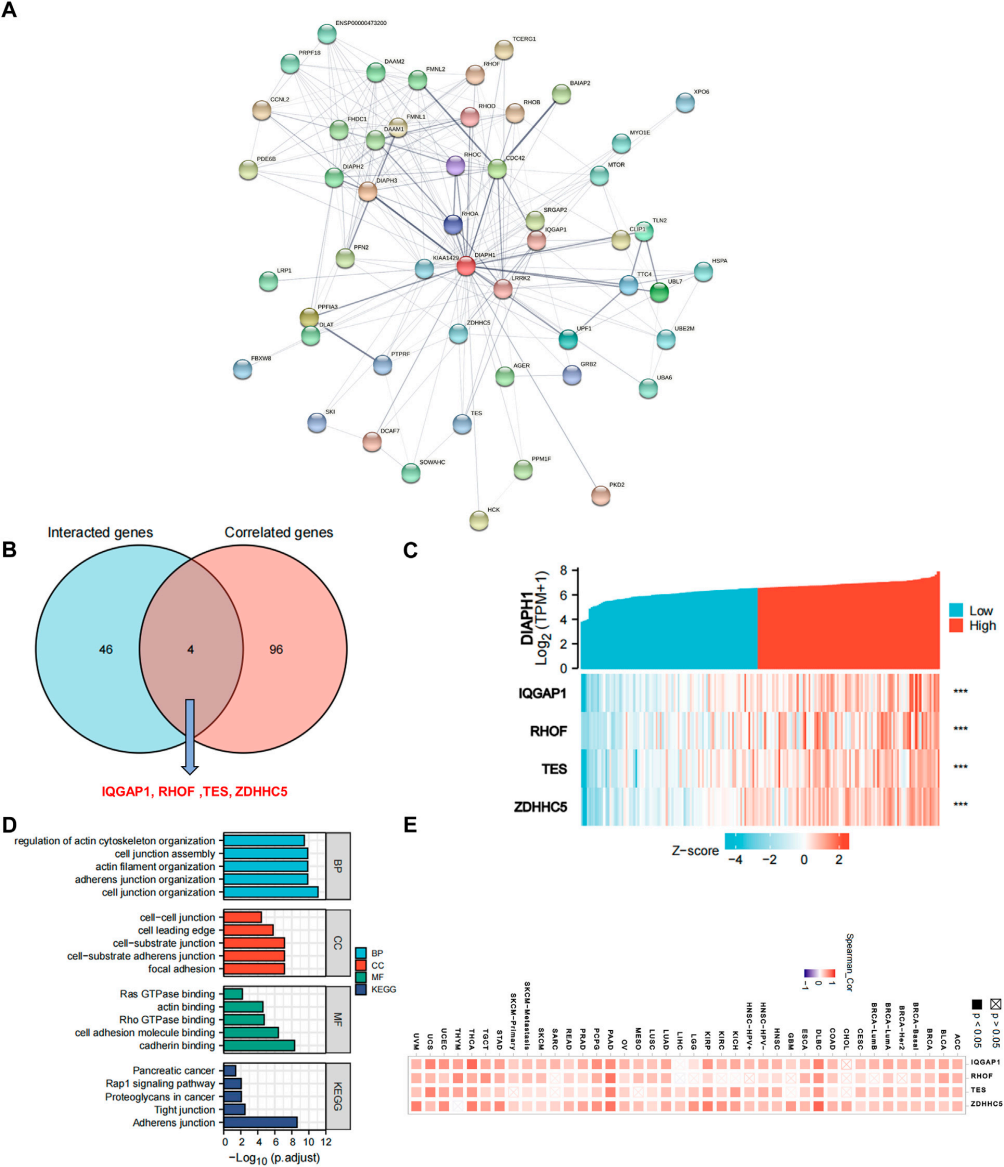
DIAPH1-related gene network, KEGG pathway analysis, and GO enrichment analysis. **(A)** A total of 50 available experimentally determined DIAPH1-interacting proteins using STRING tool. **(B)** An intersection analysis of the DIAPH1-interacting and -correlated genes. **(C)** The correlation between DIAPH1 expression and four intersected genes expression in PAAD. *** *p* < 0.001, in Spearman’s rho. TPM, transcripts per million. **(D)** GO enrichment and KEGG pathway analyses based on the DIAPH1-interacting and -correlated genes. Adjusted *p*-values were obtained from multiple hypotheses test using the Benjamini–Hochberg procedure; *p*. adjust <0.05 was considered statistically significant. **(E)** Correlation between DIAPH1 and intersected four genes expression in pan-cancer, in purity-adjusted partial Spearman’s rho.

To further explore the mechanism of DIAPHs in PAAD carcinogenesis, we conducted a series of pathway enrichment analyses for the proteins that interacted with DIAPHs and for genes that correlated with DIAPHs based on STRING tool and GEPIA2. Fifty proteins that experimentally interacted with DIAPH1 were identified in the PPI network (Figure 4A). In addition, the top 100 DIAPH1-correlated genes in PAAD of TCGA cohort were obtained (Supplementary Table S4). An intersection analysis of the genes that directly interacted with or were related to DIAPH1 identified four genes: IQ motif containing GTPase activating protein 1 (IQGAP1), Ras homolog family member F, filopodia associated (RHOF), testin LIM domain protein (TES), and zinc finger DHHC-type palmitoyltransferase 5 (ZDHHC5) (Figure 4B). The expression of these four genes in PAAD was significantly and positively associated with DIAPH1 expression (Figure 4C). Combining the two datasets, GO enrichment analysis indicated that the genes directly interacting with or related to DIAPH1 were mainly related to the BP of “cell junction organization” and “adherens junction organization,” the CC of “focal adhesion” and “cell-cell junction,” and the MF of “cadherin binding” and “cell adhesion molecule binding”. KEGG pathway analysis further suggested that the “adherens junction” and “proteoglycans in cancer” may be potential mechanisms underlying the effect of DIAPH1 on PAAD carcinogenesis (Figure 4D). In addition, the corresponding heatmap data in pan-cancer showed a significant positive correlation between DIAPH1 and the four intersecting genes in most cancer types in TCGA cohort (Figure 4E).

**FIGURE 5:**
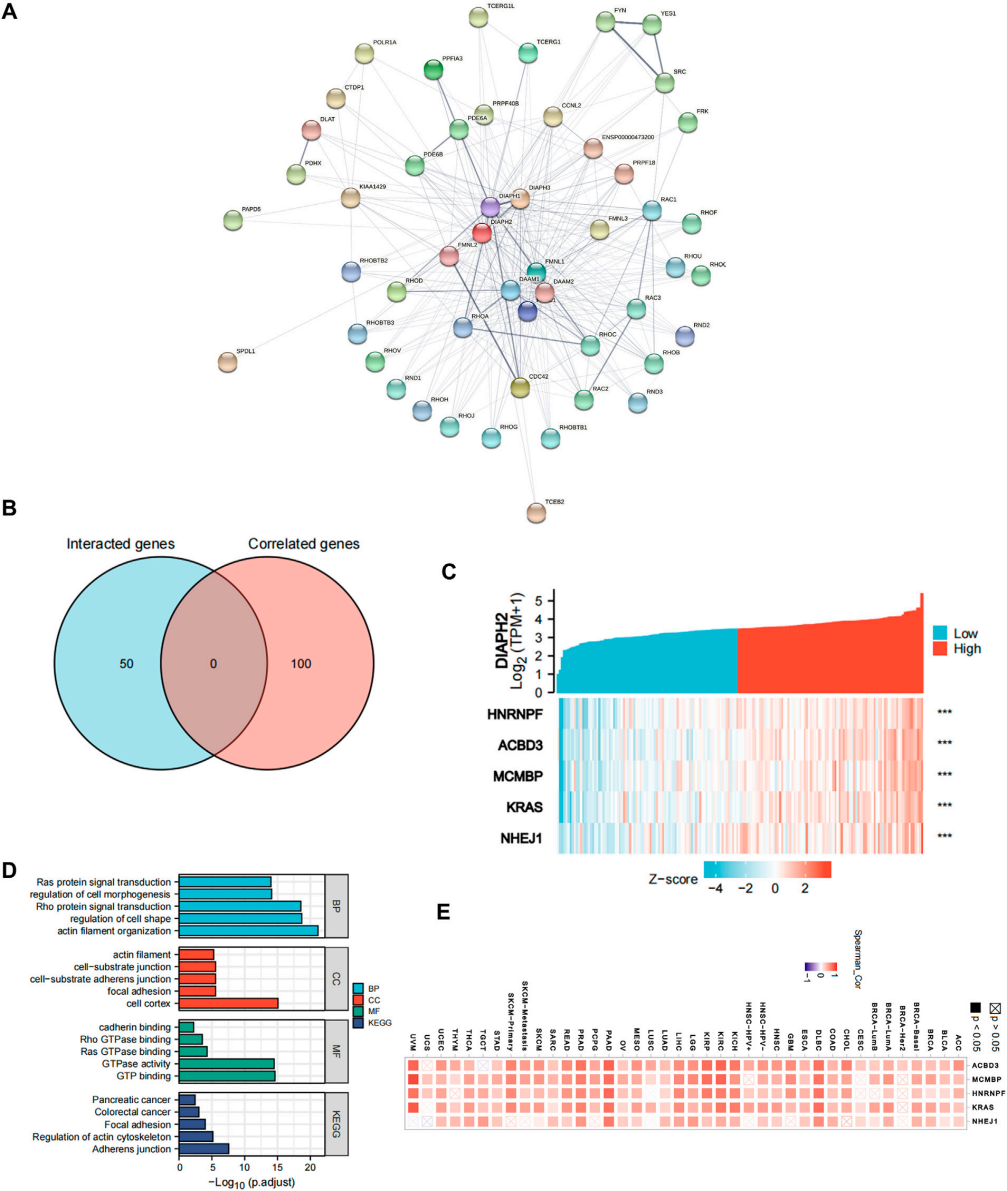
DIAPH2-related gene network, KEGG pathway analysis, and GO enrichment analysis. **(A)** A total of 50 available experimentally determined DIAPH2-interacting proteins using STRING tool. **(B)** An intersection analysis of the DIAPH2-interacting and -correlated genes. **(C)** The correlation between DIAPH2 expression and the top five DIAPH2-correlated genes expression in PAAD. *** *p* < 0.001, in Spearman’s rho. TPM, transcripts per million. **(D)** GO enrichment and KEGG pathway analyses based on the DIAPH2-interacting and -correlated genes. Adjusted *p*-values were obtained from multiple hypotheses test using the Benjamini–Hochberg procedure; *p*. adjust <0.05 was considered statistically significant. **(E)** Correlation between DIAPH2 and top five DIAPH2-correlated genes expression in pan-cancer, in purity-adjusted partial Spearman’s rho.

Fifty proteins that experimentally interacted with DIAPH2 were identified in the PPI network (Figure 5A). In addition, the top 100 DIAPH2-correlated genes in TCGA cohort were obtained (Supplementary Table S4). No genes were identified by intersection analysis of genes that directly interacted with or were related to DIAPH2 (Figure 5B). The top five DIAPH2-correlated genes in PAAD of TCGA cohort were heterogeneous nuclear ribonucleoprotein F (HNRNRF) (R = 0.76), acyl-CoA binding domain containing 3 (ACBD3) (R = 0.76), minichromosome maintenance complex binding protein (MCMBP) (R = 0.75), KRAS proto-oncogene, GTPase (KRAS) (R = 0.75), and non-homologous end joining factor 1 (NHEJ1) (R = 0.75) (Figure 5C). Combining the two datasets, GO enrichment analysis indicated that the genes directly interacting with or related to DIAPH2 were mainly related to the BP of “actin filament organization” and “regulation of cell shape,” the CC of “cell cortex” and “focal junction,” and the MF of “GTP binding” and “GTPase activity”. KEGG pathway analysis further suggested that the “adherens junction” and “regulation of actin cytoskeleton” may be potential mechanisms underlying the effect of DIAPH2 in PAAD carcinogenesis (Figure 5D). In addition, the corresponding heatmap data in pan-cancer showed a significant positive correlation between DIAPH2 and the top five DIAPH2-correlated genes of PAAD in most cancer types in TCGA cohort (Figure 5E).

**FIGURE 6:**
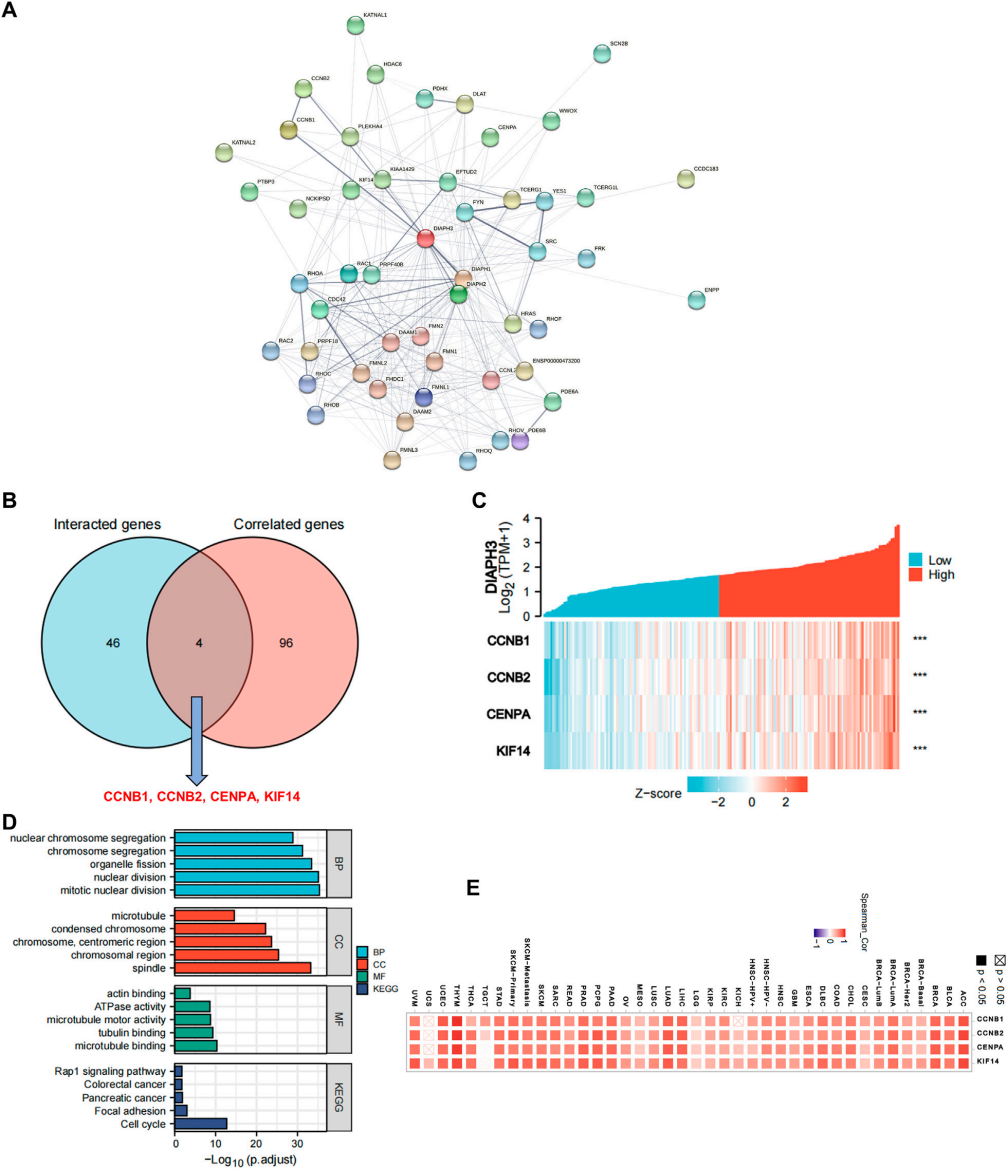
DIAPH3-related gene network, KEGG pathway analysis, and GO enrichment analysis. **(A)** A total of 50 available experimentally determined DIAPH3-interacting proteins using STRING tool. **(B)**An intersection analysis of the DIAPH3-interacting and -correlated genes. **(C)** The correlation between DIAPH3 expression and four intersected genes expression in PAAD. *** *p* < 0.001, in Spearman’s rho. TPM, transcripts per million. **(D)** GO enrichment and KEGG pathway analyses based on the DIAPH3-interacting and -correlated genes. Adjusted *p*-values were obtained from multiple hypotheses test using the Benjamini–Hochberg procedure; *p*. adjust <0.05 was considered statistically significant. **(E)** Correlation between DIAPH3 and intersected four genes expression in pan-cancer, in purity-adjusted partial Spearman’s rho.

Fifty proteins that experimentally interacted with DIAPH3 were identified in the PPI network (Figure 6A). In addition, the top 100 DIAPH3-correlated genes in TCGA cohort were obtained (Supplementary Table S4). An intersection analysis of the genes that directly interacted with or were related to DIAPH3 identified four genes: cyclin B1 (CCNB1), CCNB2, centromere protein A (CENPA), and kinesin family member 14 (KIF14) (Figure 6B). The expression of these four genes in PAAD was significantly and positively associated with DIAPH3 expression (Figure 6C). Applying the combination of the two datasets, GO enrichment analysis indicated that the genes directly interacting with or related to DIAPH3 were mainly related to the BP of “mitotic nuclear division” and “chromosome segregation,” the CC of “spindle” and “chromosomal region,” and the MF of “microtubule binding” and “tubulin binding”. KEGG pathway analysis further suggested that the “cell cycle” and “focal adhesion” may be potential mechanisms underlying the effect of DIAPH3 on PAAD carcinogenesis (Figure 6D). In addition, the corresponding heatmap data in pan-cancer showed a significant positive correlation between DIAPH3 and the four intersected four genes in most cancer types in TCGA cohort (Figure 6E).

### Gene set enrichment analysis data

**FIGURE 7:**
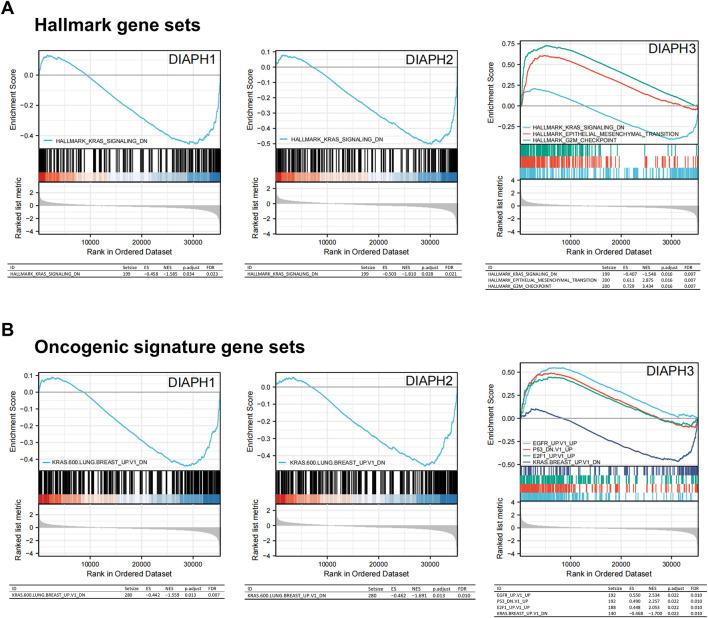
Gene set enrichment analysis of DIAPHs. **(A)** Hallmark gene sets in DIAPHs expression groups. **(B)** Oncogenic signature gene sets in DIAPHs expression groups. ES, enrichment score; NES, normalized enrichment score; FDR, false discovery rate.

The MSigDB H (hallmark gene sets) and C6 (oncogenic gene sets) databases were analyzed in the present study. The enrichment of H analysis demonstrated that the low expression of DIAPHs in PAAD of TCGA cohort was associated with genes downregulated by KRAS activation, whereas the high expression of DIAPH3 was associated with genes involved in the G2/M checkpoint and genes defining epithelial-mesenchymal transition (EMT) (Figure 7A). The enrichment of C6 analysis showed that the low expression of DIAPHs in PAAD of TCGA cohort was associated with genes downregulated by KRAS overexpression in cancer cell lines, whereas the high expression of DIAPH3 was associated with several oncogenes, such as E2F and EGFR (Figure 7B).

### Immune reactivity analysis data

**FIGURE 8:**
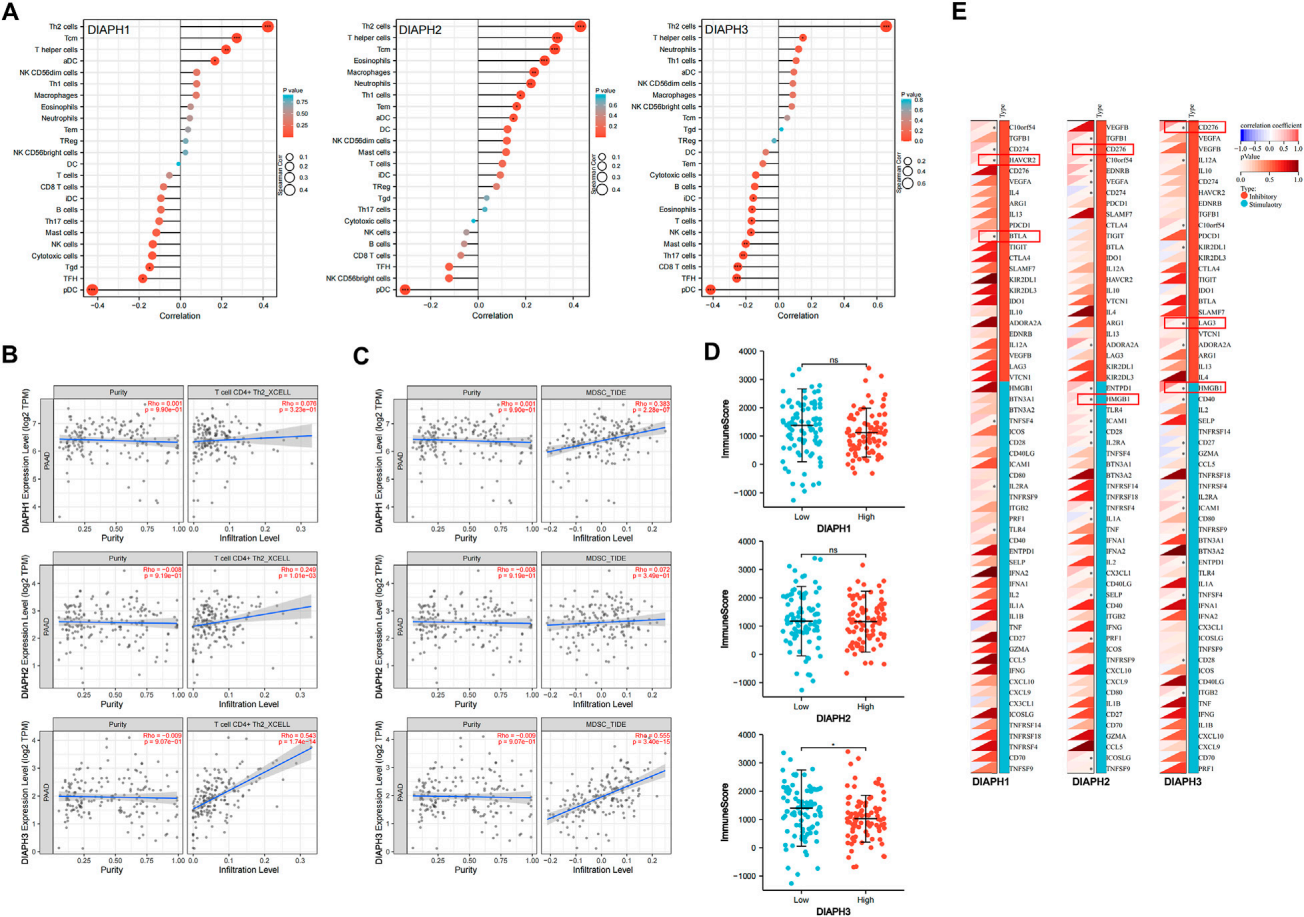
Immune reactivity analysis of DIAPHs in PAAD of TCGA database. **(A)** The correlation between DIAPHs expression and infiltration of 24 common immune cells in PAAD by ssGSEA. * *p* < 0.05, ** *p* < 0.01, *** *p* < 0.001, in Spearman’s rho. **(B)** The correlation between DIAPHs expression and infiltration of CD4^+^ Th2 cells with xCell algorithm in PAAD, in purity-adjusted Spearman’s rho. **(C)** The correlation between DIAPHs expression and infiltration of MDSCs with TIDE algorithm in PAAD, in purity-adjusted Spearman’s rho. **(D)** ImmunoScore between high- and low-expression groups based on the median expression values of DIAPHs in PAAD. * *p* < 0.05, in Wilcoxon test. **(E)** The correlation analysis of the DIAPHs expression and immune checkpoints expression in PAAD. * *p* < 0.05, in Pearson’s correlation coefficients.

**FIGURE 9:**
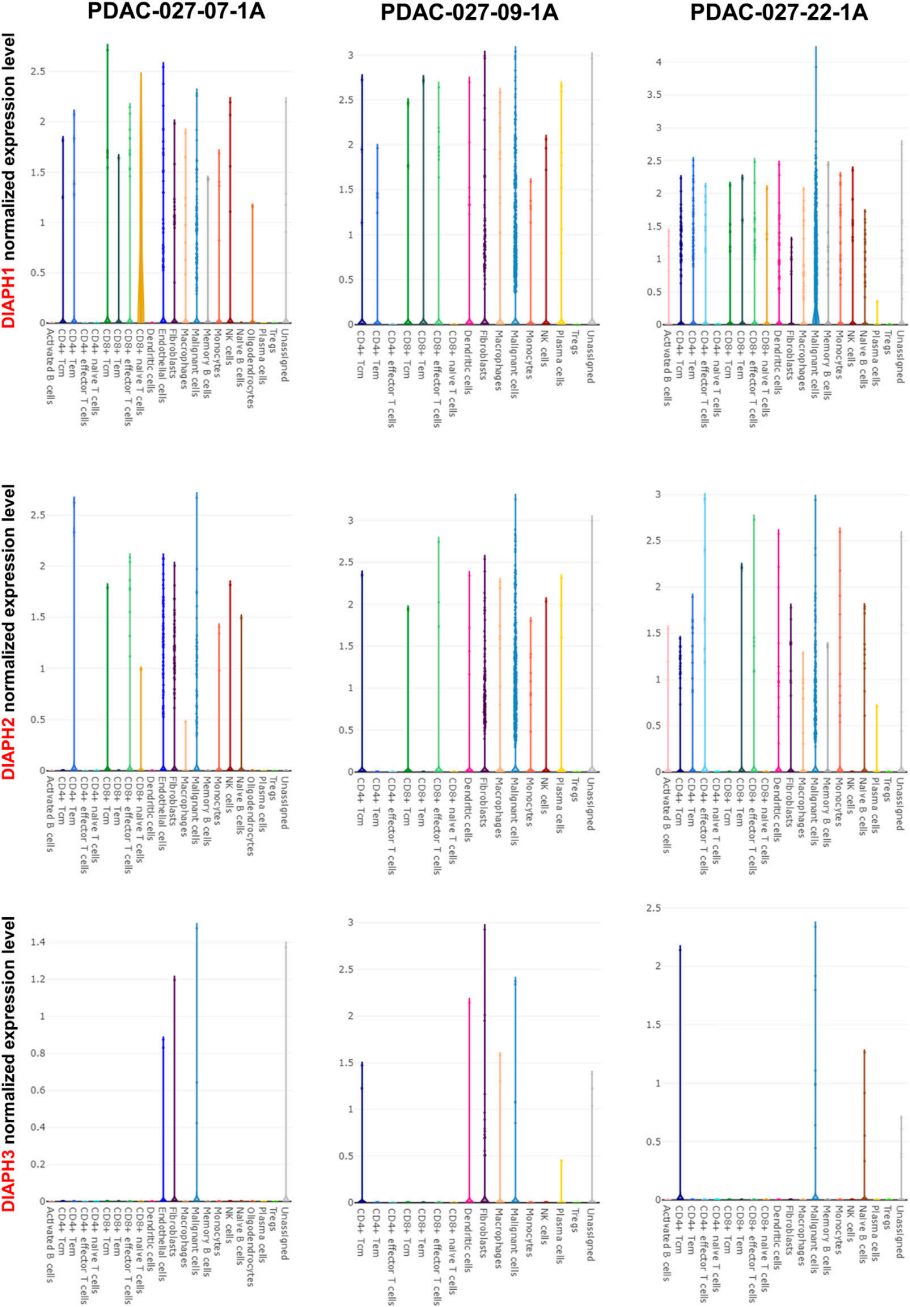
Single-cell data analysis of DIAPHs expression in PAAD. DIAPHs were mainly expressed in malignant cells and also abundantly expressed in various immune cells.

The correlation between DIAPHs expression and infiltration of 24 common immune cells in PAAD of TCGA dataset by ssGSEA showed that the expression of DIAPHs was significantly positively associated with T helper 2 (Th2) cell infiltration and significantly negatively associated with plasmacytoid dendritic cell (pDC) infiltration (Figure 8A). Furthermore, the levels of Th2 cell infiltration were significantly positively associated with DIAPH2 and DIAPH3 expression levels, as per the xCell algorithm with tumor purity adjustment (Figure 8B). In addition, significant positive correlations between the expression of DIAPH1 and DIAPH3 and the infiltration of MDSCs were observed in PAAD dataset of TCGA cohort (Figure 8C). The ImmunoScore was lower in the high DIAPH3 expression group than in the low expression group (Figure 8D). Notably, significant positive correlations between the expression of DIAPH3 and the infiltration of MDSCs were observed in all tumor types, except for THCA, UCS, GBM, and KICH in TCGA cohort (Supplementary Figure S1). Immune checkpoint analysis showed that the expression of DIAPHs in PAAD was significantly and positively correlated with immune checkpoints such as CD276, lymphocyte activating 3 (LAG3), high mobility group box 1 (HMGB1), hepatitis A virus cellular receptor 2 (HAVCR2), and B and T lymphocyte associated (BTLA) (Figure 8E). Single-cell data showed that DIAPHs was mainly expressed in malignant cells and also abundantly expressed in various immune cells in PAAD (Figure 9).

### Experimental validation

**FIGURE 10:**
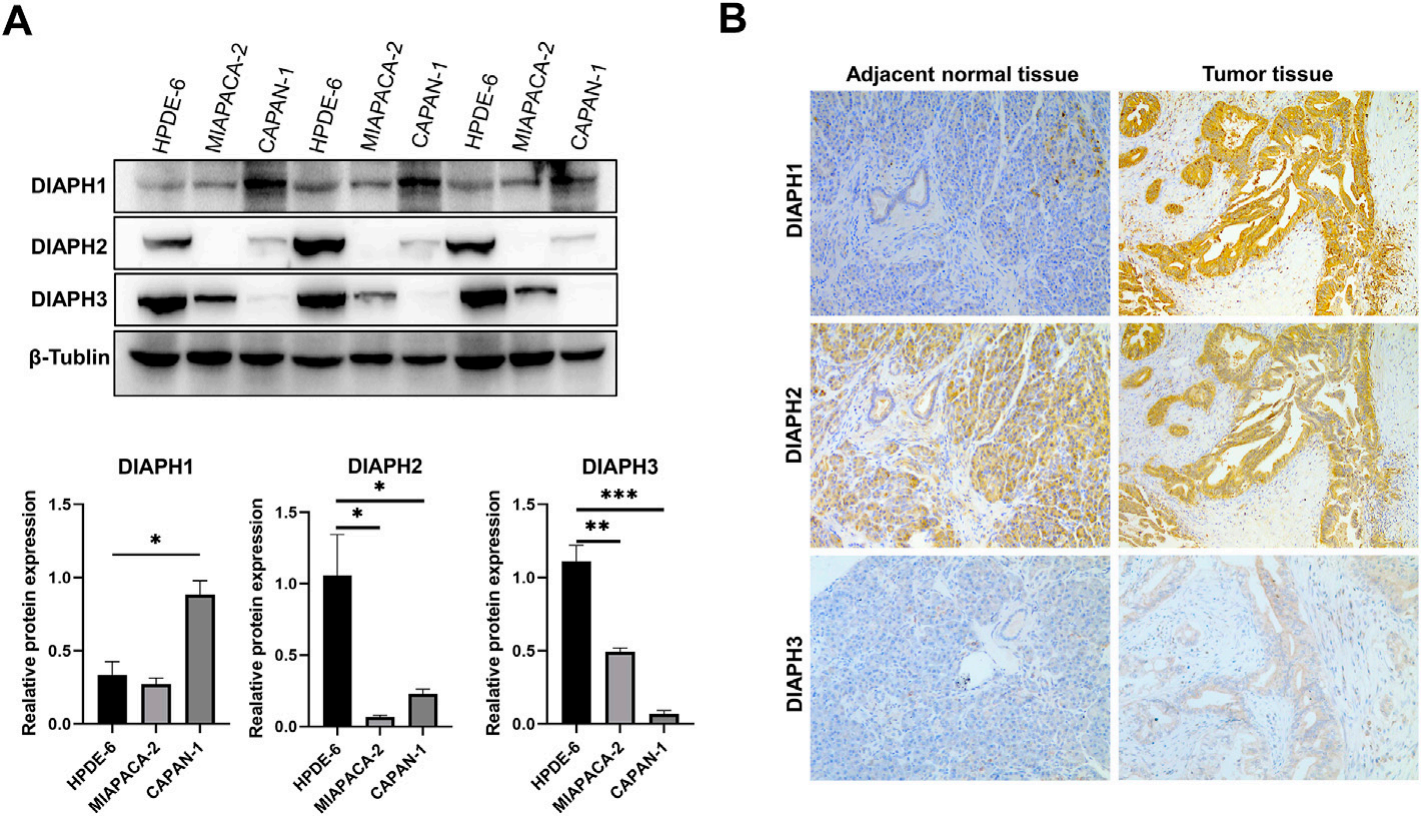
DIAPHs expression in pancreatic cancer cell lines and human pancreatic ductal adenocarcinoma. **(A)** DIAPHs expression in HPDE-6, MIAPACA-2, and CAPAN-1 cell lines. **(B)** DIAPHs expression in pancreatic ductal adenocarcinoma compared with adjacent normal tissue (×100). * *p* < 0.05, ** *p* < 0.01, *** *p* < 0.001, in Student’s *t* test.

**FIGURE 11:**
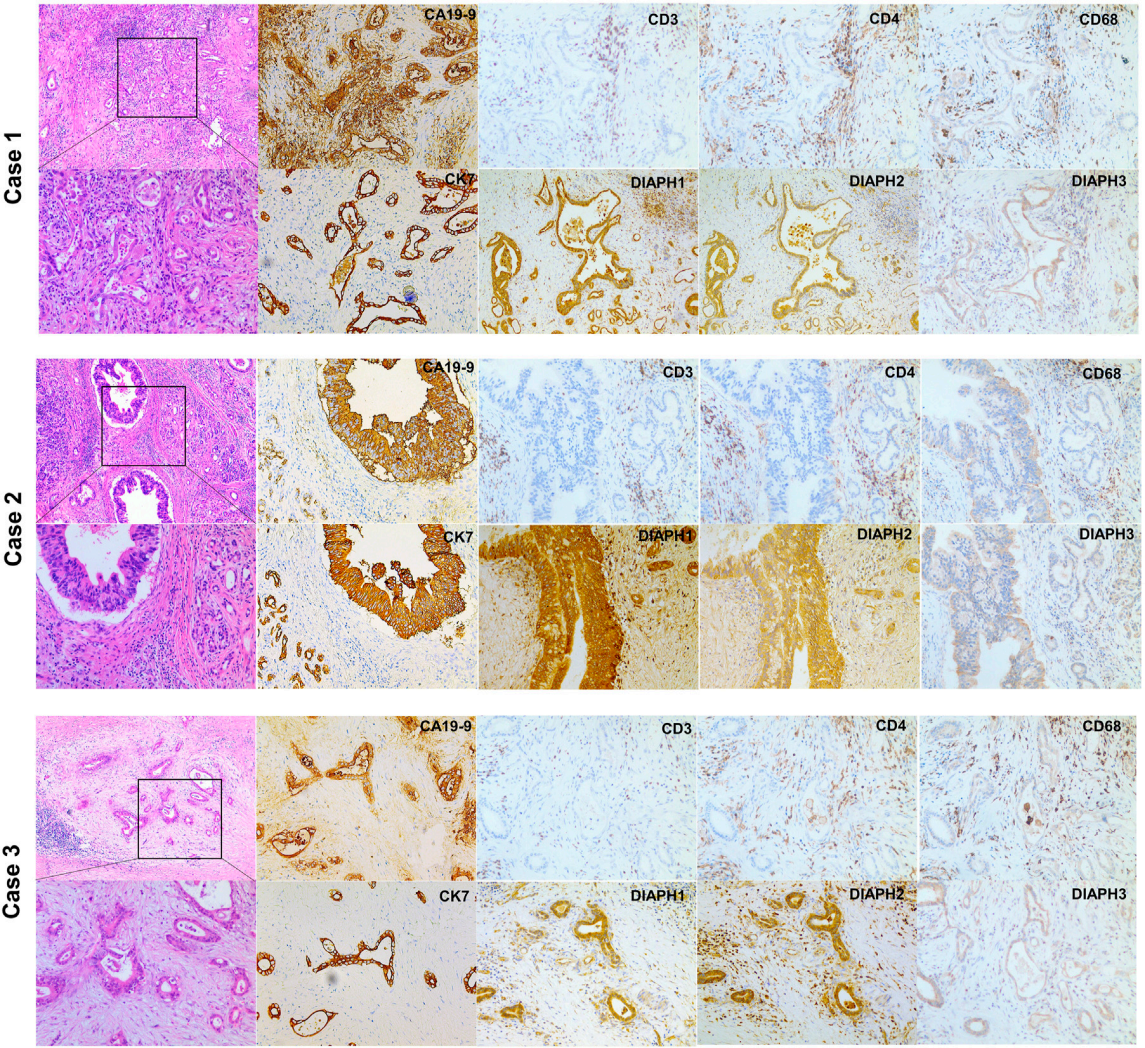
H&E and IHC staining of human pancreatic ductal adenocarcinoma. H&E staining of pancreatic ductal adenocarcinoma (left, ×40, ×100). CA19-9, CK7 and DIAPHs expression and the infiltration of T lymphocytes and macrophages in pancreatic tumor tissue (right, ×100).

The results showed that DIAPH1 is highly expressed in CAPAN-1 when compared to HPDE-6. However, DIAPH2 and DIAPH3 were poorly expressed in MIAPACA-2 and CAPAN-1 when compared to HPDE-6 (Figure 10A). The IHC data demonstrated that DIAPH1 and DIAPH3 expression were higher in tumor tissue when compared to adjacent normal tissue, and DIAPH2 was positively expressed in pancreatic acinar cells in adjacent normal tissue but not pancreatic ductal cells (Figure 10B). In addition, positive correlations between the expression of DIAPHs and the infiltration of CD3^+^ and CD4^+^ T lymphocytes, and CD68^+^ macrophages were observed in PAAD (Figure 11, Supplementary Figure S2).

## Discussion

In the present study, we demonstrated that the mRNA expression level of DIAPHs were significantly higher in PAAD tumor tissues than that in normal pancreas tissues in the consensus databases of TCGA and GTEx, as well as that total DIAPH1 and DIAPH2 protein expression levels were higher in the primary cancers than those in normal tissues for PAAD in the CPTAC dataset. In addition, high expression of DIAPHs was significantly associated with poor prognosis of PAAD in TCGA cohort. Further, KEGG pathway and GO enrichment analyses based on DIAPHs-interacting and DIAPHs-correlated genes showed that “adherens junction,” “proteoglycans in cancer,” “regulation of actin cytoskeleton,” and “cell cycle” may be the mechanisms for the effect of DIAPHs on tumorigenesis of PAAD. The expression of DIAPHs in PAAD of TCGA cohort was associated with KRAS, E2F, and EGFR signaling; positively associated with Th2 cell infiltration; and negatively associated with pDCs infiltration. Further, expression of DIAPHs in PAAD was positively correlated with CD276, LAG3, HMGB1, HAVCR2, and BTLA immune checkpoints.

DIAPH1 is an essential regulator of tissue shape change and of cells in epithelial tissues that initiate invasion (Fessenden et al., 2018). Clinical studies have shown that the expression of the DIAPH1 protein is significantly upregulated in tissues of patients with colorectal cancer and laryngeal cancer (Lin et al., 2014; Yang et al., 2021). High DIAPH1 protein levels are associated with poor prognosis in ovarian cancer patients (Schiewek et al., 2018). Several studies have demonstrated that DIAPH1 controls the metastatic capacity of colon carcinoma and glioma cells (Lin et al., 2014; Lin et al., 2015; Zhang et al., 2017). DIAPH1 upregulation inhibits apoptosis in laryngeal carcinoma cells (Yang et al., 2019). DIAPH2 expression plays an important role in invadopodia formation and is required for tumor cell invasion (Yang et al., 2007; Lizárraga et al., 2009). DIAPH2 alterations increase cellular motility and may contribute to the onset and metastasis of laryngeal cancer (Kostrzewska-Poczekaj et al., 2019; Śnit et al., 2021). To our knowledge, no clinical study has investigated the role of DIAPH2 in PAAD. DIAPH3 functions similar to those of DIAPH1 and DIAPH2, in the regulation of the remodeling cytoskeleton, and it regulates the assembly and bipolarity of the mitotic spindle (Lau et al., 2021). However, the role of DIAPH3 varies among different cancers. DIAPH3 is highly expressed in tissues of patients with pancreatic cancer, lung adenocarcinoma, and hepatocellular carcinoma (Dong et al., 2018; Xiang et al., 2019; Rong et al., 2021). DIAPH3 knockdown inhibits the proliferation of cervical cancer cells (Wan et al., 2021). In contrast, another study showed that downregulation of DIAPH3 expression is associated with metastasis in human tumors (Hager et al., 2012). DIAPH3 expression level significantly decreases in tissues of patients with breast cancer, and overexpression of DIAPH3 inhibits the migration and invasion of breast cancer cells (Jiang, 2017). The present study showed that DIAPHs expression increased significantly in PAAD in TCGA cohort and was related to poor prognosis. These data demonstrate that upregulation of DIAPHs could lead to tumorigenesis and cancer metastasis in PAAD, warranting further investigation.

Using the data for both DIAPHs-interacting proteins and DIAPHs-correlated genes, KEGG pathway analysis showed that targeting the adherens junction, regulation of the actin cytoskeleton, and cell cycle may be important mechanisms for DIAPHs in PAAD tumorigenesis. Intersection analysis of DIAPH1-interacting proteins and DIAPH1-correlated genes identified four genes (IQGAP1, RHOF, TES, and ZDHHC5) that are potentially important molecules associated with DIAPH1 in the tumorigenesis of PAAD. The interaction between DIAPH1 and IQGAP1 has been detected using an anti-bait co-immunoprecipitation assay (Brandt et al., 2007). IQGAP1 is a scaffold protein that stimulates cell motility and invasion and promotes PAAD progression (Hu et al., 2019). RHOF plays a critical role in the regulation of liver cancer metastasis (Li et al., 2021). TES is a scaffold protein that contributes to cell adhesion and cell spreading; however, it may act as a tumor suppressor in gastric cancer (Ma et al., 2010). ZDHHC5 is present in the plasma membrane and regulates cell adhesion (Woodley and Collins, 2021). To our knowledge, it is unclear whether the interactions among DIAPH1, IQGAP1, RHOF, TES, and ZDHHC5 have synergistic effects on PAAD progression. Intersection analysis of DIAPH3-interacted proteins and DIAPH3-correlated genes identified four genes (CCNB1, CCNB2, CENPA, and KIF14) that are potentially important molecules associated with DIAPH3 in the tumorigenesis of PAAD. CCNB1 and CCNB2 are essential components of cell cycle regulatory machinery. CCNB1 downregulation inhibits cell proliferation and promotes senescence in PAAD (Zhang et al., 2018). A clinical study showed that CCNB2 overexpression is a poor prognostic biomarker in patients with non-small cell lung cancer (Qian et al., 2015). CENPA is a centromere protein, and the interaction between DIAPH3 and CENPA has been detected using affinity chromatography (Yasuda et al., 2004). CENPA overexpression promotes aneuploidy and correlates with a poor prognosis in human cancers (Shrestha et al., 2021). KIF14 is a member of the kinesin superfamily of microtubule-associated motors that plays an important role in the cell cycle (Nakagawa et al., 1997). KIF14 has been identified as an oncogene in PAAD (Klimaszewska-Wiśniewska et al., 2021). The interactions among DIAPH3, CCNB1, CCNB2, CENPA, and KIF14 may contribute to PAAD progression, warranting further investigation. In this study, GSEA showed that DIAPHs expression was associated with oncogenic KRAS signaling. Furthermore, high DIAPH3 expression is associated with EMT. The mechanisms of DIAPHs in PAAD may involve KRAS signaling and EMT.

Considering the increasing significance of tumor microenvironment (TME), cancer research has increasingly focused on immune infiltration and immunotherapy (Sanmamed and Chen, 2018). However, the role of DIAPHs in immunity remains unclear. A recent study showed that loss of DIAPH1 causes immunodeficiency (Kaustio et al., 2021). Cytoskeletal remodeling mediated by DIAPH1 is essential for T cell and DC immune responses (Eisenmann et al., 2007; Tanizaki et al., 2010). In the present study, DIAPHs expression was positively associated with Th2 cell infiltration, whereas it was negatively associated with pDC infiltration. In addition, our experimental validation showed that positive correlations between the expression of DIAPH3 and the infiltration of CD3^+^ and CD4^+^ T lymphocytes, and CD68^+^ macrophages were observed in PAAD. Liu and others demonstrated that CD4^+^ T cells, but not CD8^+^ T cells, are essential for tissue healing and remodeling of the blood vasculature in cancer. The host-directed protective response is dependent on the Th2 cytokine interleukin-4 (IL-4), which indicates that type 2 immunity could be an effective tissue-level defense mechanism against cancer (Liu et al., 2020). Intratumoral Th2 cell infiltration correlates with a poor prognosis in patients with PAAD (De Monte et al., 2011). pDCs play a key role in the induction and maintenance of antitumor immunity (Palucka and Banchereau, 2012). A clinical study found that higher densities of intratumor pDCs were associated with prolonged survival in patients with colon cancer (Kießler et al., 2021). DC paucity leads to dysfunctional immune surveillance in PAAD (Hegde et al., 2020). Recent studies showed that IGFBP2 signaling pathway contributes to the macrophage-based immunosuppressive microenvironment in PAAD (Sun et al., 2021). Notably, DIAPH3 expression was positively associated with MDSC infiltration in PAAD of TCGA cohort. ImmunoScore was lower in the high DIAPH3 expression group than in the low expression group. MDSCs are immunomodulatory cells that suppress adaptive immune responses and promote tumor progression (Veglia et al., 2021). Specifically, depletion of MDSCs in PAAD increases the intratumoral accumulation of CD8^+^ T cells and apoptosis of tumor cells (Stromnes et al., 2014). The correlation among DIAPHs expression and Th2 cell, pDC, and MDSC infiltration in PAAD requires further investigation.

Immunotherapy is an emerging cancer treatment that helps the immune system fight tumors. Among the most attractive approaches to activate therapeutic antitumor immunity is blocking immune checkpoints (Pardoll, 2012). In the present study, the expression of DIAPHs in PAAD was positively correlated with immune checkpoints such as CD276, LAG3, and HMGB1. A clinical study reported that CD276 expression was significantly higher in PAAD tissues than in normal tissues and was associated with poor prognosis (Yamato et al., 2009). LAG3 expression by infiltrating T cells correlates with poor prognosis in patients with PAAD (Seifert et al., 2021). HMGB1 plays both oncogenic and tumor-suppressive roles in tumor development (Kang et al., 2013). Diminished HMGB1 expression in patients with PAAD correlates with poor overall survival (Kang et al., 2017). A large proportion of patients with positive initial responses may develop resistance to the immune checkpoint inhibitors (Schoenfeld and Hellmann, 2020). Two small-molecule intramimics (IMM-01 and IMM-02) of DIAPHs can slow tumor growth by targeting the cytoskeletal remodeling machinery in cancer cells (Lash et al., 2013). The combination of small-molecule drugs for DIAPHs and immune checkpoint blockers may be an effective option for the treatment of PAAD.

In conclusion, the present study revealed that DIAPHs could serve as novel prognostic biomarkers and TME regulators of PAAD. These data may provide important information regarding the role and mechanisms of DIAPHs in tumorigenesis and PAAD immunotherapy.

## Data Availability

The original contributions presented in the study are included in the article/Supplementary Material, further inquiries can be directed to the corresponding author.

## References

[B1] Bailey P. Chang D. K. Nones K. Johns A. L. Patch A. M. Gingras M. C. (2016). Genomic analyses identify molecular subtypes of pancreatic cancer. Nature 531 (7592), 47–52. 10.1038/nature16965 26909576

[B2] Biankin A. V. Waddell N. Kassahn K. S. Gingras M. C. Muthuswamy L. B. Johns A. L. (2012). Pancreatic cancer genomes reveal aberrations in axon guidance pathway genes. Nature 491 (7424), 399–405. 10.1038/nature11547 23103869 PMC3530898

[B3] Bindea G. Mlecnik B. Tosolini M. Kirilovsky A. Waldner M. Obenauf A. C. (2013). Spatiotemporal dynamics of intratumoral immune cells reveal the immune landscape in human cancer. Immunity 39 (4), 782–795. 10.1016/j.immuni.2013.10.003 24138885

[B4] Brandt D. T. Marion S. Griffiths G. Watanabe T. Kaibuchi K. Grosse R. (2007). Dia1 and IQGAP1 interact in cell migration and phagocytic cup formation. J. Cell Biol. 178 (2), 193–200. 10.1083/jcb.200612071 17620407 PMC2064439

[B5] Cangkrama M. Liu H. Whipman J. Zubair M. Matsushita M. Di Filippo M. (2022). A protumorigenic mDia2-MIRO1 Axis controls mitochondrial positioning and function in cancer-associated fibroblasts. Cancer Res. 82 (20), 3701–3717. 10.1158/0008-5472.CAN-22-0162 35997559 PMC9574377

[B6] Cangkrama M. Wietecha M. Mathis N. Okumura R. Ferrarese L. Al-Nuaimi D. (2020). A paracrine activin A-mDia2 axis promotes squamous carcinogenesis via fibroblast reprogramming. EMBO Mol. Med. 12 (4), e11466. 10.15252/emmm.201911466 32150356 PMC7136968

[B7] Cao L. Huang C. Cui Zhou D. Hu Y. Lih T. M. Savage S. R. (2021). Proteogenomic characterization of pancreatic ductal adenocarcinoma. Cell 184 (19), 5031–5052.e26. e26. 10.1016/j.cell.2021.08.023 34534465 PMC8654574

[B8] Chen F. Chandrashekar D. S. Varambally S. Creighton C. J. (2019). Pan-cancer molecular subtypes revealed by mass-spectrometry-based proteomic characterization of more than 500 human cancers. Nat. Commun. 10 (1), 5679. 10.1038/s41467-019-13528-0 31831737 PMC6908580

[B9] Cheng L. Zhang J. Ahmad S. Rozier L. Yu H. Deng H. (2011). Aurora B regulates formin mDia3 in achieving metaphase chromosome alignment. Dev. Cell 20 (3), 342–352. 10.1016/j.devcel.2011.01.008 21397845 PMC4581877

[B10] Chesarone M. A. DuPage A. G. Goode B. L. (2010). Unleashing formins to remodel the actin and microtubule cytoskeletons. Nat. Rev. Mol. Cell Biol. 11 (1), 62–74. 10.1038/nrm2816 19997130

[B11] De Monte L. Reni M. Tassi E. Clavenna D. Papa I. Recalde H. (2011). Intratumor T helper type 2 cell infiltrate correlates with cancer-associated fibroblast thymic stromal lymphopoietin production and reduced survival in pancreatic cancer. J. Exp. Med. 208 (3), 469–478. 10.1084/jem.20101876 21339327 PMC3058573

[B12] Dong L. Li Z. Xue L. Li G. Zhang C. Cai Z. (2018). DIAPH3 promoted the growth, migration and metastasis of hepatocellular carcinoma cells by activating beta-catenin/TCF signaling. Mol. Cell. Biochem. 438 (1-2), 183–190. 10.1007/s11010-017-3125-7 28795316

[B13] Eisenmann K. M. West R. A. Hildebrand D. Kitchen S. M. Peng J. Sigler R. (2007). T cell responses in mammalian diaphanous-related formin mDia1 knock-out mice. J. Biol. Chem. 282 (34), 25152–25158. 10.1074/jbc.M703243200 17595162

[B14] Fessenden T. B. Beckham Y. Perez-Neut M. Ramirez-San Juan G. Chourasia A. H. Macleod K. F. (2018). Dia1-dependent adhesions are required by epithelial tissues to initiate invasion. J. Cell Biol. 217 (4), 1485–1502. 10.1083/jcb.201703145 29437785 PMC5881494

[B15] Fischer A. H. Jacobson K. A. Rose J. Zeller R. (2008). Hematoxylin and eosin staining of tissue and cell sections. CSH Protoc. 2008, prot4986. 10.1101/pdb.prot4986:pdb. 21356829

[B16] Gao J. Aksoy B. A. Dogrusoz U. Dresdner G. Gross B. Sumer S. O. (2013). Integrative analysis of complex cancer genomics and clinical profiles using the cBioPortal. Sci. Signal. 6 (269), pl1. 10.1126/scisignal.2004088 23550210 PMC4160307

[B17] Hager M. H. Morley S. Bielenberg D. R. Gao S. Morello M. Holcomb I. N. (2012). DIAPH3 governs the cellular transition to the amoeboid tumour phenotype. EMBO Mol. Med. 4 (8), 743–760. 10.1002/emmm.201200242 22593025 PMC3494074

[B18] Hänzelmann S. Castelo R. Guinney J. (2013). Gsva: Gene set variation analysis for microarray and RNA-seq data. BMC Bioinforma. 14, 7. 10.1186/1471-2105-14-7 PMC3618321 23323831

[B19] Hegde S. Krisnawan V. E. Herzog B. H. Zuo C. Breden M. A. Knolhoff B. L. (2020). Dendritic cell paucity leads to dysfunctional immune surveillance in pancreatic cancer. Cancer Cell 37 (3), 289–307. e9. 10.1016/j.ccell.2020.02.008 32183949 PMC7181337

[B20] Hu W. Wang Z. Zhang S. Lu X. Wu J. Yu K. (2019). IQGAP1 promotes pancreatic cancer progression and epithelial-mesenchymal transition (EMT) through Wnt/β-catenin signaling. Sci. Rep. 9 (1), 7539. 10.1038/s41598-019-44048-y 31101875 PMC6525164

[B21] Iasonos A. Schrag D. Raj G. V. Panageas K. S. (2008). How to build and interpret a nomogram for cancer prognosis. J. Clin. Oncol. 26 (8), 1364–1370. 10.1200/JCO.2007.12.9791 18323559

[B22] Jiang J. (2017). Diaphanous-related formin-3 overexpression inhibits the migration and invasion of triple-negative breast cancer by inhibiting RhoA-GTP expression. Biomed. Pharmacother. 94, 439–445. 10.1016/j.biopha.2017.07.119 28779705

[B23] Kang R. Xie Y. Zhang Q. Hou W. Jiang Q. Zhu S. (2017). Intracellular HMGB1 as a novel tumor suppressor of pancreatic cancer. Cell Res. 27 (7), 916–932. 10.1038/cr.2017.51 28374746 PMC5518983

[B24] Kang R. Zhang Q. Zeh H. J. 3rd Lotze M. T. Tang D. (2013). HMGB1 in cancer: Good, bad, or both? Clin. Cancer Res. 19 (15), 4046–4057. 10.1158/1078-0432.CCR-13-0495 23723299 PMC3732559

[B25] Katoh M. Katoh M. (2004). Identification and characterization of human DIAPH3 gene *in silico* . Int. J. Mol. Med. 13 (3), 473–478. 10.3892/ijmm.13.3.473 14767582

[B26] Kaustio M. Nayebzadeh N. Hinttala R. Tapiainen T. Åström P. Mamia K. (2021). Loss of DIAPH1 causes SCBMS, combined immunodeficiency, and mitochondrial dysfunction. J. Allergy Clin. Immunol. 148 (2), 599–611. 10.1016/j.jaci.2020.12.656 33662367

[B27] Kießler M. Plesca I. Sommer U. Wehner R. Wilczkowski F. Müller L. (2021). Tumor-infiltrating plasmacytoid dendritic cells are associated with survival in human colon cancer. J. Immunother. Cancer 9 (3), e001813. 10.1136/jitc-2020-001813 33762320 PMC7993360

[B28] Klein A. P. (2021). Pancreatic cancer epidemiology: Understanding the role of lifestyle and inherited risk factors. Nat. Rev. Gastroenterol. Hepatol. 18 (7), 493–502. 10.1038/s41575-021-00457-x 34002083 PMC9265847

[B29] Klimaszewska-Wiśniewska A. Neska-Długosz I. Buchholz K. Durślewicz J. Grzanka D. Kasperska A. (2021). Prognostic significance of KIF11 and KIF14 expression in pancreatic adenocarcinoma. Cancers (Basel) 13 (12), 3017. 10.3390/cancers13123017 34208606 PMC8234517

[B30] Kostrzewska-Poczekaj M. Byzia E. Soloch N. Jarmuz-Szymczak M. Janiszewska J. Kowal E. (2019). DIAPH2 alterations increase cellular motility and may contribute to the metastatic potential of laryngeal squamous cell carcinoma. Carcinogenesis 40 (10), 1251–1259. 10.1093/carcin/bgz035 30793164

[B31] Lammers M. Meyer S. Kühlmann D. Wittinghofer A. (2008). Specificity of interactions between mDia isoforms and Rho proteins. J. Biol. Chem. 283 (50), 35236–35246. 10.1074/jbc.M805634200 18829452 PMC3259862

[B32] Lash L. L. Wallar B. J. Turner J. D. Vroegop S. M. Kilkuskie R. E. Kitchen-Goosen S. M. (2013). Small-molecule intramimics of formin autoinhibition: A new strategy to target the cytoskeletal remodeling machinery in cancer cells. Cancer Res. 73 (22), 6793–6803. 10.1158/0008-5472.CAN-13-1593 24242070

[B33] Lau E. O. Damiani D. Chehade G. Ruiz-Reig N. Saade R. Jossin Y. (2021). DIAPH3 deficiency links microtubules to mitotic errors, defective neurogenesis, and brain dysfunction. Elife 10, e61974. 10.7554/eLife.61974 33899739 PMC8102060

[B34] Li S. Liu Y. Bai Y. Chen M. Cheng D. Wu M. (2021). Ras homolog family member F, filopodia associated promotes hepatocellular carcinoma metastasis by altering the metabolic status of cancer cells through RAB3D. Hepatology 73 (6), 2361–2379. 10.1002/hep.31641 33205519

[B35] Lin Y. N. Bhuwania R. Gromova K. Failla A. V. Lange T. Riecken K. (2015). Drosophila homologue of Diaphanous 1 (DIAPH1) controls the metastatic potential of colon cancer cells by regulating microtubule-dependent adhesion. Oncotarget 6 (21), 18577–18589. 10.18632/oncotarget.4094 26124177 PMC4621911

[B36] Lin Y. N. Izbicki J. R. König A. Habermann J. K. Blechner C. Lange T. (2014). Expression of DIAPH1 is up-regulated in colorectal cancer and its down-regulation strongly reduces the metastatic capacity of colon carcinoma cells. Int. J. Cancer 134 (7), 1571–1582. 10.1002/ijc.28486 24105619

[B37] Liu J. Lichtenberg T. Hoadley K. A. Poisson L. M. Lazar A. J. Cherniack A. D. (2018). An integrated TCGA pan-cancer clinical data resource to drive high-quality survival outcome analytics. Cell 173 (2), 400–416. e11. 10.1016/j.cell.2018.02.052 29625055 PMC6066282

[B38] Liu M. Kuo F. Capistrano K. J. Kang D. Nixon B. G. Shi W. (2020). TGF-β suppresses type 2 immunity to cancer. Nature 587 (7832), 115–120. 10.1038/s41586-020-2836-1 33087928 PMC8347705

[B39] Lizárraga F. Poincloux R. Romao M. Montagnac G. Le Dez G. Bonne I. (2009). Diaphanous-related formins are required for invadopodia formation and invasion of breast tumor cells. Cancer Res. 69 (7), 2792–2800. 10.1158/0008-5472.CAN-08-3709 19276357

[B40] Lynch E. D. Lee M. K. Morrow J. E. Welcsh P. L. León P. E. King M. C. (1997). Nonsyndromic deafness DFNA1 associated with mutation of a human homolog of the Drosophila gene diaphanous. Science 278 (5341), 1315–1318. 10.1126/science.278.5341.1315 9360932

[B41] Ma H. Weng D. Chen Y. Huang W. Pan K. Wang H. (2010). Extensive analysis of D7S486 in primary gastric cancer supports TESTIN as a candidate tumor suppressor gene. Mol. Cancer 9, 190. 10.1186/1476-4598-9-190 20626849 PMC2915979

[B42] Nakagawa T. Tanaka Y. Matsuoka E. Kondo S. Okada Y. Noda Y. (1997). Identification and classification of 16 new kinesin superfamily (KIF) proteins in mouse genome. Proc. Natl. Acad. Sci. U. S. A. 94 (18), 9654–9659. 10.1073/pnas.94.18.9654 9275178 PMC23244

[B43] Palazzo A. F. Cook T. A. Alberts A. S. Gundersen G. G. (2001). mDia mediates Rho-regulated formation and orientation of stable microtubules. Nat. Cell Biol. 3 (8), 723–729. 10.1038/35087035 11483957

[B44] Palucka K. Banchereau J. (2012). Cancer immunotherapy via dendritic cells. Nat. Rev. Cancer 12 (4), 265–277. 10.1038/nrc3258 22437871 PMC3433802

[B45] Pardoll D. M. (2012). The blockade of immune checkpoints in cancer immunotherapy. Nat. Rev. Cancer 12 (4), 252–264. 10.1038/nrc3239 22437870 PMC4856023

[B46] Qian X. Song X. He Y. Yang Z. Sun T. Wang J. (2015). CCNB2 overexpression is a poor prognostic biomarker in Chinese NSCLC patients. Biomed. Pharmacother. 74, 222–227. 10.1016/j.biopha.2015.08.004 26349989

[B47] Robin X. Turck N. Hainard A. Tiberti N. Lisacek F. Sanchez J. C. (2011). pROC: an open-source package for R and S+ to analyze and compare ROC curves. BMC Bioinforma. 12, 77. 10.1186/1471-2105-12-77 PMC3068975 21414208

[B48] Rong Y. Gao J. Kuang T. Chen J. Li J. A. Huang Y. (2021). DIAPH3 promotes pancreatic cancer progression by activating selenoprotein TrxR1-mediated antioxidant effects. J. Cell. Mol. Med. 25 (4), 2163–2175. 10.1111/jcmm.16196 33345387 PMC7882936

[B49] Sanmamed M. F. Chen L. (2018). A paradigm shift in cancer immunotherapy: From enhancement to normalization. Cell 175 (2), 313–326. 10.1016/j.cell.2018.09.035 30290139 PMC6538253

[B50] Schiewek J. Schumacher U. Lange T. Joosse S. A. Wikman H. Pantel K. (2018). Clinical relevance of cytoskeleton associated proteins for ovarian cancer. J. Cancer Res. Clin. Oncol. 144 (11), 2195–2205. 10.1007/s00432-018-2710-9 30094535 PMC11813493

[B51] Schoenfeld A. J. Hellmann M. D. (2020). Acquired resistance to immune checkpoint inhibitors. Cancer Cell 37 (4), 443–455. 10.1016/j.ccell.2020.03.017 32289269 PMC7182070

[B52] Seifert L. Plesca I. Müller L. Sommer U. Heiduk M. von Renesse J. (2021). LAG-3-Expressing tumor-infiltrating T cells are associated with reduced disease-free survival in pancreatic cancer. Cancers (Basel) 13 (6), 1297. 10.3390/cancers13061297 33803936 PMC7998134

[B53] Shrestha R. L. Rossi A. Wangsa D. Hogan A. K. Zaldana K. S. Suva E. (2021). CENP-A overexpression promotes aneuploidy with karyotypic heterogeneity. J. Cell Biol. 220 (4), e202007195. 10.1083/jcb.202007195 33620383 PMC7905998

[B54] Śnit M. Misiołek M. Ścierski W. Koniewska A. Stryjewska-Makuch G. Okła S. (2021). DIAPH2, PTPRD and HIC1 gene polymorphisms and laryngeal cancer risk. Int. J. Environ. Res. Public Health 18 (14), 7486. 10.3390/ijerph18147486 34299935 PMC8305316

[B55] Stromnes I. M. Brockenbrough J. S. Izeradjene K. Carlson M. A. Cuevas C. Simmons R. M. (2014). Targeted depletion of an MDSC subset unmasks pancreatic ductal adenocarcinoma to adaptive immunity. Gut 63 (11), 1769–1781. 10.1136/gutjnl-2013-306271 24555999 PMC4340484

[B56] Sun L. Zhang X. Song Q. Liu L. Forbes E. Tian W. (2021). IGFBP2 promotes tumor progression by inducing alternative polarization of macrophages in pancreatic ductal adenocarcinoma through the STAT3 pathway. Cancer Lett. 500, 132–146. 10.1016/j.canlet.2020.12.008 33309859 PMC7923838

[B57] Tang Z. Kang B. Li C. Chen T. Zhang Z. (2019). GEPIA2: An enhanced web server for large-scale expression profiling and interactive analysis. Nucleic Acids Res. 47 (W1), W556–W560. 10.1093/nar/gkz430 31114875 PMC6602440

[B58] Tanizaki H. Egawa G. Inaba K. Honda T. Nakajima S. Moniaga C. S. (2010). Rho-mDia1 pathway is required for adhesion, migration, and T-cell stimulation in dendritic cells. Blood 116 (26), 5875–5884. 10.1182/blood-2010-01-264150 20881208

[B59] Veglia F. Sanseviero E. Gabrilovich D. I. (2021). Myeloid-derived suppressor cells in the era of increasing myeloid cell diversity. Nat. Rev. Immunol. 21 (8), 485–498. 10.1038/s41577-020-00490-y 33526920 PMC7849958

[B60] Vivian J. Rao A. A. Nothaft F. A. Ketchum C. Armstrong J. Novak A. (2017). Toil enables reproducible, open source, big biomedical data analyses. Nat. Biotechnol. 35 (4), 314–316. 10.1038/nbt.3772 28398314 PMC5546205

[B61] Wan L. Zhu J. Wu Q. (2021). Knockdown of DIAPH3 inhibits the proliferation of cervical cancer cells through inactivating mTOR signaling pathway. J. Oncol. 2021, 4228241. 10.1155/2021/4228241 34659408 PMC8514916

[B62] Witkiewicz A. K. McMillan E. A. Balaji U. Baek G. Lin W. C. Mansour J. (2015). Whole-exome sequencing of pancreatic cancer defines genetic diversity and therapeutic targets. Nat. Commun. 6, 6744. 10.1038/ncomms7744 25855536 PMC4403382

[B63] Woodley K. T. Collins M. O. (2021). Regulation and function of the palmitoyl-acyltransferase ZDHHC5. Febs J. 288 (23), 6623–6634. 10.1111/febs.15709 33415776

[B64] Xiang G. Weiwei H. Erji G. Haitao M. (2019). DIAPH3 promotes the tumorigenesis of lung adenocarcinoma. Exp. Cell Res. 385 (1), 111662. 10.1016/j.yexcr.2019.111662 31586548

[B65] Yamana N. Arakawa Y. Nishino T. Kurokawa K. Tanji M. Itoh R. E. (2006). The Rho-mDia1 pathway regulates cell polarity and focal adhesion turnover in migrating cells through mobilizing Apc and c-Src. Mol. Cell. Biol. 26 (18), 6844–6858. 10.1128/MCB.00283-06 16943426 PMC1592856

[B66] Yamato I. Sho M. Nomi T. Akahori T. Shimada K. Hotta K. (2009). Clinical importance of B7-H3 expression in human pancreatic cancer. Br. J. Cancer 101 (10), 1709–1716. 10.1038/sj.bjc.6605375 19844235 PMC2778545

[B67] Yang C. Czech L. Gerboth S. Kojima S. Scita G. Svitkina T. (2007). Novel roles of formin mDia2 in lamellipodia and filopodia formation in motile cells. PLoS Biol. 5 (11), e317. 10.1371/journal.pbio.0050317 18044991 PMC2229861

[B68] Yang J. Huang Q. Guo Y. Wei Z. Zhou L. Chen H. (2021). DIAPH1 promotes laryngeal squamous cell carcinoma progression through cell cycle regulation. Front. Oncol. 11, 716876. 10.3389/fonc.2021.716876 34631544 PMC8494199

[B69] Yang J. Zhou L. Zhang Y. Zheng J. Zhou J. Wei Z. (2019). DIAPH1 is upregulated and inhibits cell apoptosis through ATR/p53/Caspase-3 signaling pathway in laryngeal squamous cell carcinoma. Dis. Markers 2019, 6716472. 10.1155/2019/6716472 30733838 PMC6348834

[B70] Yasuda S. Oceguera-Yanez F. Kato T. Okamoto M. Yonemura S. Terada Y. (2004). Cdc42 and mDia3 regulate microtubule attachment to kinetochores. Nature 428 (6984), 767–771. 10.1038/nature02452 15085137

[B71] Yoshihara K. Shahmoradgoli M. Martínez E. Vegesna R. Kim H. Torres-Garcia W. (2013). Inferring tumour purity and stromal and immune cell admixture from expression data. Nat. Commun. 4, 2612. 10.1038/ncomms3612 24113773 PMC3826632

[B72] Yu G. Wang L. G. Han Y. He Q. Y. (2012). clusterProfiler: an R package for comparing biological themes among gene clusters. Omics 16 (5), 284–287. 10.1089/omi.2011.0118 22455463 PMC3339379

[B73] Zeng J. Zhang Y. Shang Y. Mai J. Shi S. Lu M. (2022). CancerSCEM: A database of single-cell expression map across various human cancers. Nucleic Acids Res. 50 (D1), D1147–D1155. 10.1093/nar/gkab905 34643725 PMC8728207

[B74] Zhang C. Wang L. Chen J. Liang J. Xu Y. Li Z. (2017). Knockdown of Diaph1 expression inhibits migration and decreases the expression of MMP2 and MMP9 in human glioma cells. Biomed. Pharmacother. 96, 596–602. 10.1016/j.biopha.2017.10.031 29035824

[B75] Zhang H. Zhang X. Li X. Meng W. B. Bai Z. T. Rui S. Z. (2018). Effect of CCNB1 silencing on cell cycle, senescence, and apoptosis through the p53 signaling pathway in pancreatic cancer. J. Cell. Physiol. 234 (1), 619–631. 10.1002/jcp.26816 30069972

