# Supplementary material for: Diaphanous-related formin subfamily: Novel prognostic biomarkers and tumor microenvironment regulators for pancreatic adenocarcinoma
Source: Front Mol Biosci. 2022 Dec 15;9:910950. doi: 10.3389/fmolb.2022.910950 (PMC9797685; doi:10.3389/fmolb.2022.910950)

**Table S1**. Baseline of age and TNM stages between low- and high-DIAPH1 expression group in PAAD.

| Characteristic | Low expression of **DIAPH1** | High expression of **DIAPH1** | p |
| --- | --- | --- | --- |
| n | 89 | 89 |  |
| T stage, n (%) |  |  | 0.465 |
| T1 | 4 (2.3%) | 3 (1.7%) |  |
| T2 | 15 (8.5%) | 9 (5.1%) |  |
| T3 | 66 (37.5%) | 76 (43.2%) |  |
| T4 | 2 (1.1%) | 1 (0.6%) |  |
| N stage, n (%) |  |  | 0.829 |
| N0 | 26 (15%) | 24 (13.9%) |  |
| N1 | 60 (34.7%) | 63 (36.4%) |  |
| M stage, n (%) |  |  | 0.647 |
| M0 | 33 (39.3%) | 46 (54.8%) |  |
| M1 | 3 (3.6%) | 2 (2.4%) |  |
| Age, mean ± SD | 65.55 ± 10.35 | 63.94 ± 11.23 | 0.322 |

**Table S2.** Baseline of age and TNM stages between low- and high-DIAPH2 expression group in PAAD.

| Characteristic | Low expression of **DIAPH2** | High expression of **DIAPH2** | p |
| --- | --- | --- | --- |
| n | 89 | 89 |  |
| T stage, n (%) |  |  | 0.143 |
| T1 | 3 (1.7%) | 4 (2.3%) |  |
| T2 | 15 (8.5%) | 9 (5.1%) |  |
| T3 | 66 (37.5%) | 76 (43.2%) |  |
| T4 | 3 (1.7%) | 0 (0%) |  |
| N stage, n (%) |  |  | 0.488 |
| N0 | 22 (12.7%) | 28 (16.2%) |  |
| N1 | 63 (36.4%) | 60 (34.7%) |  |
| M stage, n (%) |  |  | 0.363 |
| M0 | 39 (46.4%) | 40 (47.6%) |  |
| M1 | 1 (1.2%) | 4 (4.8%) |  |
| Age, mean ± SD | 64.65 ± 10.81 | 64.84 ± 10.84 | 0.906 |

**Table S3.** Baseline of age and TNM stages between low- and high-DIAPH3 expression group in PAAD.

| Characteristic | Low expression of **DIAPH3** | High expression of **DIAPH3** | p |
| --- | --- | --- | --- |
| n | 89 | 89 |  |
| T stage, n (%) |  |  | 0.810 |
| T1 | 4 (2.3%) | 3 (1.7%) |  |
| T2 | 13 (7.4%) | 11 (6.2%) |  |
| T3 | 68 (38.6%) | 74 (42%) |  |
| T4 | 2 (1.1%) | 1 (0.6%) |  |
| N stage, n (%) |  |  | 0.754 |
| N0 | 26 (15%) | 24 (13.9%) |  |
| N1 | 59 (34.1%) | 64 (37%) |  |
| M stage, n (%) |  |  | 1.000 |
| M0 | 36 (42.9%) | 43 (51.2%) |  |
| M1 | 2 (2.4%) | 3 (3.6%) |  |
| Age, mean ± SD | 65.36 ± 10.77 | 64.13 ± 10.85 | 0.451 |

**Table S4.** DIAPHs correlated and interacted genes based on String, and GEPIA2.

| DIAPH1 |  | DIAPH2 |  | DIAPH3 |  |
| --- | --- | --- | --- | --- | --- |
| Interacted genes | Correlated genes | Interacted genes | Correlated genes | Interacted genes | Correlated genes |
| AGER | CTNNA1 | CCNL2 | HNRNPF | CCDC183 | CKAP2L |
| BAIAP2 | WDR55 | CDC42 | ACBD3 | CCNB1 | TPX2 |
| CCNL2 | MGAT4B | CTDP1 | MCMBP | CCNB2 | BUB1 |
| CDC42 | TMBIM1 | DAAM1 | KRAS | CCNL2 | SKA3 |
| CLIP1 | PCDH1 | DAAM2 | NHEJ1 | CDC42 | DLGAP5 |
| DAAM1 | TNIP1 | DIAPH1 | ABI1 | CENPA | MCM4 |
| DAAM2 | F11R | DIAPH3 | GCC2 | DAAM1 | ANLN |
| DCAF7 | TNFRSF10A | DLAT | ZDHHC9 | DAAM2 | CENPF |
| DIAPH2 | MVP | ENSP00000473200 | SEC23IP | DIAPH1 | WDHD1 |
| DIAPH3 | DNM2 | FHDC1 | GPD2 | DIAPH2 | KIF23 |
| DLAT | KCNK1 | FMNL1 | KLF3 | DLAT | ASPM |
| ENSP00000473200 | CTNND1 | FMNL2 | BFAR | EFTUD2 | GINS1 |
| FBXW8 | ZDHHC5 | FMNL3 | PRKAA1 | ENPP6 | KIF14 |
| FHDC1 | STK24 | FRK | VPS35 | ENSP00000473200 | HMMR |
| FMNL1 | TOR4A | FYN | SEPT10 | FHDC1 | CENPE |
| FMNL2 | PATL1 | KIAA1429 | STRN | FMN1 | KIF20A |
| GRB2 | SMAD3 | PAPD5 | SPOPL | FMN2 | PRR11 |
| HCK | CHP1 | PDE6A | TOX4 | FMNL1 | MCM2 |
| HSPA4L | ATG16L1 | PDE6B | PGGT1B | FMNL2 | CEP55 |
| IQGAP1 | ADGRE5 | PDHX | SMNDC1 | FMNL3 | NEK2 |
| KIAA1429 | LIPH | POLR1A | RBM27 | FRK | KIAA1524 |
| LRP1 | CAPN2 | PPFIA3 | PTBP3 | FYN | CHEK1 |
| LRRK2 | ZDHHC7 | PRPF18 | STAU1 | HDAC6 | HJURP |
| MTOR | APH1A | PRPF40B | AGPS | HRAS | CCNA2 |
| MYO1E | RPS6KA4 | RAC1 | PRPF40A | KATNAL1 | SGOL1 |
| PDE6B | RELA | RAC2 | GNL3L | KATNAL2 | DTL |
| PFN2 | LRRC1 | RAC3 | SLC44A1 | KIAA1429 | TTK |
| PKD2 | BFAR | RHOA | SPTY2D1 | KIF14 | UHRF1 |
| PPFIA3 | SH3RF2 | RHOB | RAB8A | NCKIPSD | SGOL2 |
| PPM1F | TMEM92 | RHOBTB1 | DDX18 | PDE6A | KIF4A |
| PRPF18 | ZBTB7A | RHOBTB2 | CSNK1G3 | PDE6B | KIF18A |
| PTPRF | MARK2 | RHOBTB3 | ZC3H15 | PDHX | KIF20B |
| RHOA | B3GNT3 | RHOC | TBC1D8B | PLEKHA4 | NUSAP1 |
| RHOB | SH3RF1 | RHOD | MAT2B | PRPF18 | KIF11 |
| RHOC | ARHGEF5 | RHOF | ACTR3 | PRPF40B | CDK1 |
| RHOD | PARD6B | RHOG | PPP3R1 | PTBP3 | C1orf112 |
| RHOF | CGN | RHOH | GORASP2 | RAC1 | NCAPG2 |
| SKI | CTTN | RHOJ | CAPRIN1 | RAC2 | CKAP2 |
| SOWAHC | HARS2 | RHOQ | OTUD7B | RHOA | MAD2L1 |
| SRGAP2 | BTBD10 | RHOU | CASK | RHOB | DEPDC1 |
| TCERG1 | ZBTB7B | RHOV | CAPZA2 | RHOC | NUF2 |
| TES | ZDHHC3 | RND1 | MBNL3 | RHOF | KIF2C |
| TLN2 | TMEM63B | RND2 | UEVLD | RHOQ | RACGAP1 |
| TTC4 | VDAC1 | RND3 | PRRC1 | RHOV | RFC3 |
| UBA6 | SLK | SPDL1 | ARL5B | SCN2B | INCENP |
| UBE2M | GATAD2A | SRC | CHMP3 | SRC | NCAPH |
| UBL7 | CCDC120 | TCEB2 | SPRED1 | TCERG1 | AURKA |
| UPF1 | PELI1 | TCERG1 | ACTR2 | TCERG1L | MCM10 |
| XPO6 | HECTD1 | TCERG1L | RBM7 | WWOX | NDC1 |
| ZDHHC5 | LDLR | YES1 | PDZD8 | YES1 | SMC4 |
|  | AGFG1 |  | FEM1C |  | MZT1 |
|  | CAPN1 |  | CRIM1 |  | ORC1 |
|  | POLD4 |  | MPZL1 |  | ERCC6L |
|  | SRC |  | CERS6 |  | CENPI |
|  | CORO2A |  | ERLIN1 |  | ORC6 |
|  | MPZL3 |  | ZFP91 |  | SHCBP1 |
|  | IQGAP1 |  | YIPF4 |  | MTBP |
|  | SDC4 |  | ROCK2 |  | RAD51 |
|  | BAZ1A |  | SLC25A43 |  | AUNIP |
|  | ST14 |  | SLAIN2 |  | BUB1B |
|  | HNRNPAB |  | FBXO34 |  | PLK4 |
|  | FA2H |  | ATRX |  | ZWILCH |
|  | ARL6IP1 |  | RAB3GAP1 |  | BORA |
|  | LONRF3 |  | ADD3 |  | NEIL3 |
|  | TJP2 |  | YME1L1 |  | GMPS |
|  | KCTD5 |  | SP3 |  | CLSPN |
|  | CNNM4 |  | TCF7L2 |  | CENPN |
|  | LAD1 |  | DCTN5 |  | DSCC1 |
|  | EFNB1 |  | POF1B |  | ECT2 |
|  | KLF3 |  | EIF3A |  | FAM64A |
|  | OSBPL2 |  | STK26 |  | E2F7 |
|  | CLINT1 |  | DYNC1LI2 |  | BRIP1 |
|  | OSBPL3 |  | ARHGAP42 |  | FAM72D |
|  | UBE2I |  | CD2AP |  | CCNB1 |
|  | AAMP |  | TRAF6 |  | KIF18B |
|  | EPB41L1 |  | DDX21 |  | NUP155 |
|  | BCL10 |  | SDHD |  | ZNF367 |
|  | CTDSP1 |  | ATP11B |  | PRIM2 |
|  | ASAP2 |  | CTTNBP2NL |  | CTD-2510F5.4 |
|  | SLC10A3 |  | CAB39 |  | NCAPG |
|  | STK39 |  | YY1 |  | PAICS |
|  | HNRNPF |  | KIF5B |  | RAD51AP1 |
|  | TUFT1 |  | ACER3 |  | CDC6 |
|  | ZDHHC9 |  | G3BP1 |  | CENPK |
|  | PRELID2 |  | WDR44 |  | ASF1B |
|  | TMC7 |  | FCHO2 |  | CCNB2 |
|  | F2RL1 |  | HNRNPK |  | FOXM1 |
|  | CDH1 |  | YIPF6 |  | EXO1 |
|  | ABTB2 |  | TM9SF3 |  | FAM72B |
|  | ARF6 |  | TRIM44 |  | CDCA2 |
|  | C1orf116 |  | HNRNPU |  | ARHGAP11A |
|  | SMG7 |  | ELF4 |  | CDKN3 |
|  | PNP |  | TROVE2 |  | KIFC1 |
|  | TES |  | SRFBP1 |  | GINS2 |
|  | ELF4 |  | CKAP5 |  | MCM6 |
|  | TAGLN2 |  | COPS2 |  | ZWINT |
|  | LMO7 |  | CTNND1 |  | CENPA |
|  | LGALS3 |  | ARHGAP5 |  | MELK |
|  | PLEKHA7 |  | C2orf49 |  | SPDL1 |
|  | RHOF |  | RSF1 |  | CDCA4 |

**Figure S1.** Correlation between DIAPH3 expression and MDSCs infiltraion in pan-cancer.
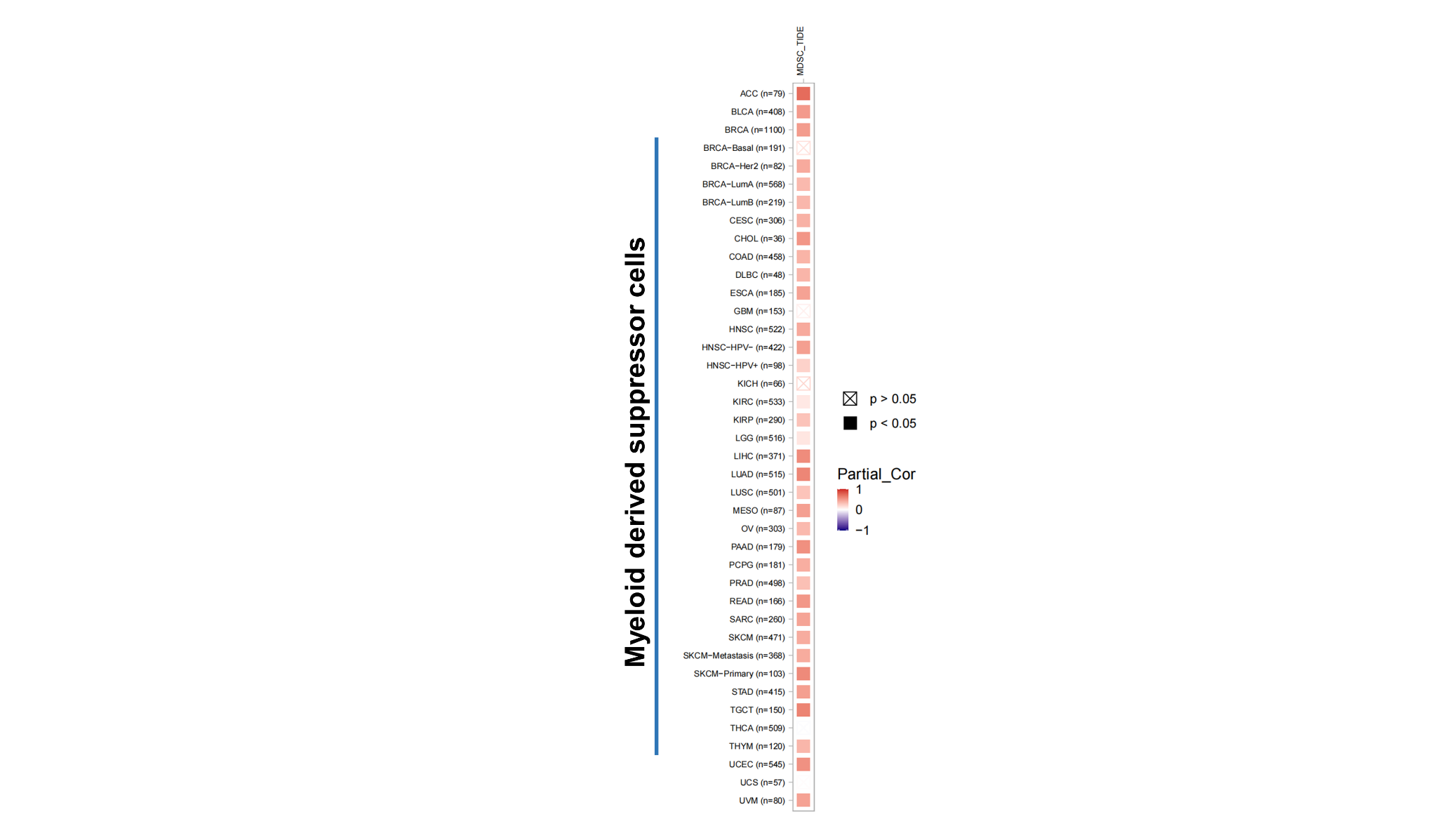


**Figure S2.** H&E staining of pancreatic ductal adenocarcinoma (left, ×40, ×100). CA19-9, CK7 and DIAPH3 expression and the infiltration of T lymphocytes and macrophage in corresponding sections (right, ×100).


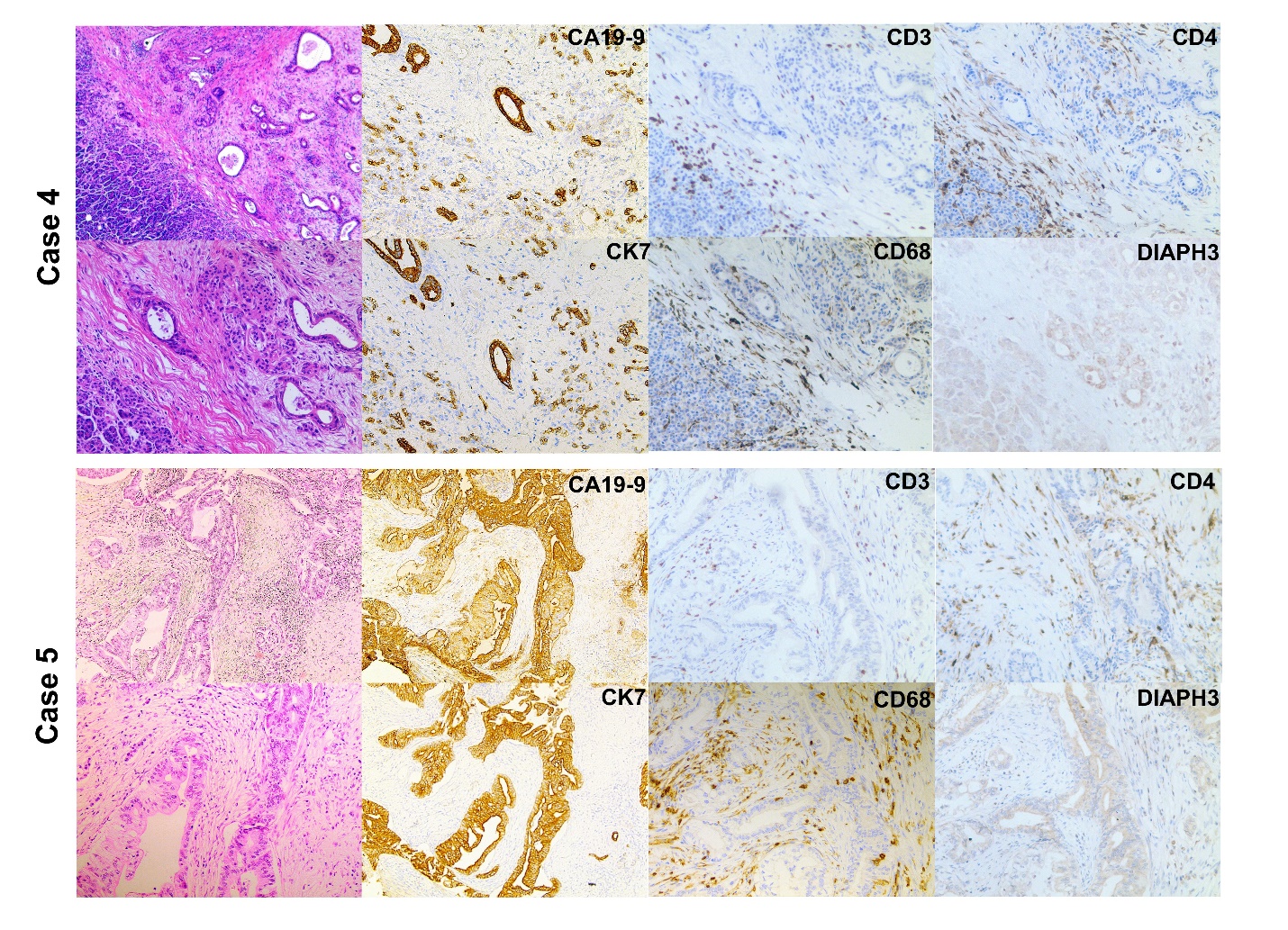

Supplement: Supplementary file 1 [file Table1.DOCX]
